# An Autoantigen Atlas from Human Lung HFL1 Cells Offers Clues to Neurological and Diverse Autoimmune Manifestations of COVID-19

**DOI:** 10.1101/2021.01.24.427965

**Published:** 2021-01-24

**Authors:** Julia Y. Wang, Wei Zhang, Michael W. Roehrl, Victor B. Roehrl, Michael H. Roehrl

**Affiliations:** 1 Curandis, New York, USA; 2 Department of Gastroenterology, Affiliated Hospital of Guizhou Medical University, Guizhou, China; 3 Department of Pathology, Memorial Sloan Kettering Cancer Center, New York, USA; 4 Human Oncology and Pathogenesis Program, Memorial Sloan Kettering Cancer Center, New York, USA

**Keywords:** COVID-19, SARS-Cov-2, autoantigens, autoantibodies, dermatan sulfate, autoimmunity

## Abstract

COVID-19 is accompanied by a myriad of both transient and long-lasting autoimmune responses. Dermatan sulfate (DS), a glycosaminoglycan crucial for wound healing, has unique affinity for autoantigens (autoAgs) from apoptotic cells. DS-autoAg complexes are capable of stimulating autoreactive B cells and autoantibody production. Using DS affinity, we identified an autoantigenome of 408 proteins from human fetal lung fibroblast HFL11 cells, at least 231 of which are known autoAgs. Comparing with available COVID data, 352 proteins of the autoantigenome have thus far been found to be altered at protein or RNA levels in SARS-Cov-2 infection, 210 of which are known autoAgs. The COVID-altered proteins are significantly associated with RNA metabolism, translation, vesicles and vesicle transport, cell death, supramolecular fibrils, cytoskeleton, extracellular matrix, and interleukin signaling. They offer clues to neurological problems, fibrosis, smooth muscle dysfunction, and thrombosis. In particular, 150 altered proteins are related to the nervous system, including axon, myelin sheath, neuron projection, neuronal cell body, and olfactory bulb. An association with the melanosome is also identified. The findings from our study illustrate a strong connection between viral infection and autoimmunity. The vast number of COVID-altered proteins with propensity to become autoAgs offers an explanation for the diverse autoimmune complications in COVID patients. The variety of autoAgs related to mRNA metabolism, translation, and vesicles raises concerns about potential adverse effects of mRNA vaccines. The COVID autoantigen atlas we are establishing provides a detailed molecular map for further investigation of autoimmune sequelae of the pandemic.

## Summary sentence

An autoantigenome by dermatan sulfate affinity from human lung HFL1 cells may explain neurological and autoimmune manifestations of COVID-19

## Introduction

The emergence of the novel coronavirus SARS-CoV-2 has dragged the world into a prolonged pandemic. Aside from the intensively studied ACE2, heparan sulfate is another crucial entry receptor for coronaviruses (1). Dermatan sulfate (DS), structurally and functionally similar to heparan sulfate and heparin, belongs to the glycosaminoglycan family. Many viruses, including Ebola, Vaccinia, Zika, Dengue, and Hepatitis C viruses, have been shown to interact with glycosaminoglycans (2–5). These polyanionic polysaccharides consist of disaccharide repeating units of amino sugars and uronic acids with varying degrees of sulfation. Glycosaminoglycans are major components of the extracellular matrix and basement membrane, act as a filler between cells and tissue fibers and have numerous biological functions.

DS is most abundant in the skin but is also found in lungs, blood vessels, heart valves, and tendons. DS plays important roles in cell death, wound healing, and tissue repair. In human wound fluid, DS is the most abundant glycosaminoglycan (6). Its biosynthesis is increased by fibroblasts, epithelial cells, and capillary endothelial cells in wounded skin, mucosal ulcers, and inflammation-associated angiogenesis (7–9). Its molecular size also changes during wound healing, with elongated DS polymers packing along thin collagen fibrils in wounded skin (10). After tissue injury, fibroblasts require DS to migrate from the stroma surrounding the injury into the fibrin-laden wound to facilitate granulation tissue formation and wound healing (11).

DS is also a key molecule in autoimmunity, as we have discovered (12–16). DS is the most potent among glycosaminoglycans in stimulating autoreactive B1 cells and autoantibody production (12, 13). DS has a peculiar affinity to apoptotic cells and their released autoantigens (autoAgs), and macromolecular autoAg-DS affinity complexes are capable of engaging autoBCRs in a dual signaling event to activate B1 cells (13, 14). Recently, we also found that DS may steer autoreactive B1 cell fate at the pre-B stage by regulating the immunoglobulin heavy chain of the precursor BCR (17). Our studies illustrate a unifying property of autoAgs, i.e., self-molecules with DS affinity have a high propensity to become autoAgs, which explains how seemingly unrelated self-molecules can all induce humoral autoimmunity via similar immunological signaling events. In support of our hypothesis and by using DS affinity, we have cataloged hundreds of classic and novel autoAgs (14–16, 18).

A diverse spectrum of autoimmune symptoms has been observed in COVID-19 patients, including autoimmune cytopenia, multisystem inflammatory syndrome in children, immune-mediated neurological syndromes, Guillain-Barré syndrome, connective tissue disease-associated interstitial lung disease, antiphospholipid syndrome, autoimmune hemolytic anemia, autoimmune encephalitis, systemic lupus erythematosus, optic neuritis and myelitis, and acquired hemophilia (19–26). Many autoantibodies have been identified in COVID patients, including ANA (antinuclear antibody), ENA (extractable nuclear antigen), ANCA (anti-neutrophil cytoplasmic antibody), lupus anticoagulant, antiphospholipid, anti-IFN, anti-myelin oligodendrocyte glycoprotein, and antiheparin-PF4 complex (19–27).

To understand autoimmune sequelae of COVID, we aimed to establish a COVID autoantigen atlas that will serve as a molecular map to guide further investigation. In this study, we identified an autoantigenome of 408 proteins from human fetal lung fibroblast HFL1 cells by DS-affinity fractionation and protein sequencing, with at least 231 being known autoAgs. We then compared these with currently available data from SARS-CoV-2-infected patients and cells (as of 12/14/2020 in Coronascape) (28–48). Remarkably, 352 (86.3%) of these proteins have been found to be altered (up- or down-regulated) at protein and/or RNA expression levels, and 210 of the COVID-altered proteins are known autoAgs in a great variety of autoimmune diseases and cancers. The COVID-altered proteins reveal intricate host responses to the viral infection and point to close associations with diverse disease manifestations of COVID-19.

## Results and Discussion

### An autoantigenome of 408 proteins with DS-affinity from HFL1 cells

Proteins extracted from HFL1 cells were fractionated with DS-affinity resins. The DS-binding fraction eluting with 0.5 M NaCl yielded 306 proteins by mass spectrometry sequencing, corresponding to proteins with medium-to-strong DS affinity. The fraction eluting with 1.0 M NaCl yielded 121 proteins, corresponding to proteins with very strong DS affinity. After excluding redundancies, a total of 408 unique proteins were obtained (Table 1). To verify how many of these proteins are known autoAgs, we conducted an extensive literature search for autoantibodies specific for each protein. Remarkably, at least 231 (56.6%) of our DS-affinity proteins already have known associated specific autoantibodies in various diseases and are thus confirmed autoAgs (see references in Table 1).

Of those not yet confirmed as autoAgs, a majority are similar to known autoAgs. As an example, we identified 18 ribosomal proteins, of which 9 have been individually identified as autoAgs (Table 1); however, anti-ribosomal autoantibodies are reported to react with a heterogeneous pool of many ribosomal proteins (49). Therefore, many of the ribosomal proteins we identified may be true but yet-to-be-confirmed autoAgs. As another example, autoantibodies against the 20S proteasome core are reported to be polyspecific and react with many subunits (50). Thus, although only 7 of 15 proteasome proteins we identified are thus far individually confirmed, the remainder may be true but yet-to-be-specified autoAgs. Similarly, some members of eukaryotic translation initiation and elongation factors are confirmed autoAgs, while others await confirmation. In summary, the putative autoantigenome from HFL1 cells provides at least 231 confirmed and 177 yet-to-confirm putative autoAgs (Table 1).

### DS-affinity proteins are functionally connected and enriched

**Fig. 1.**
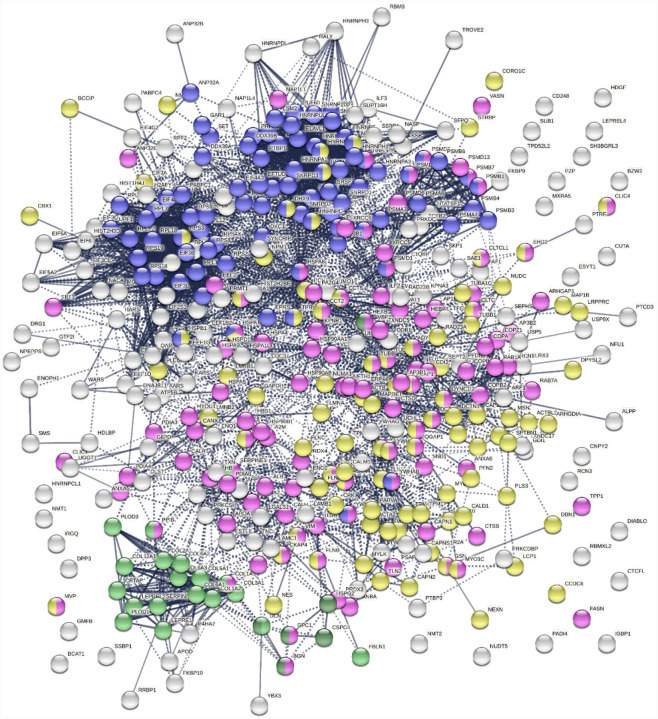
The 408-protein autoantigenome identified by DS-affinity from HFL1 cells forms a highly interacting network. Connecting lines represent interactions with high confidence (minimum interaction score of 0.7) as per STRING analysis. Colored proteins are involved in metabolism of RNA (blue), vesicles (pink), cytoskeleton (gold), collagen and elastic fibers (light green), and chondroitin sulfate/dermatan sulfate metabolism (dark green).

To find out whether DS-affinity-associated proteins are a random collection or biologically connected, we performed protein-protein interaction analyses with STRING (51). Of the 408 DS-associated proteins, 405 proteins recognized by STRING (ANP32C, ANXA2P2, HSP90AA2 excluded) have 7,582 interactions, whereas a random set of 405 proteins is expected to have only 3,060 interactions; hence, DS-affinity proteins represent a significantly connected network with PPI enrichment p-value <1.0E-6 (Fig. 1). Based on cellular component classification, these proteins are highly concentrated in the nucleus (226 proteins), vesicles (111 proteins), ribonucleoprotein complexes (95 proteins), and the cytoskeleton (95 proteins).

Pathway and process analyses by STRING and Metascape (28) revealed that the mRNA metabolic process is the most enriched GO Biological Process, and the top KEGG pathways are the spliceosome and protein processing in the endoplasmic reticulum. The top Reactome pathways are metabolism of RNA, metabolism of proteins, and axon guidance. The top local network clusters are GTP hydrolysis and joining of the 60S ribosomal subunits and mRNA splicing. The Molecular Complex Detection algorithm identified clusters related to eukaryotic translation elongation, cellular responses to stress, regulation of RNA stability, COPI-independent Golgi-to-ER retrograde traffic, and supramolecular fiber organization.

### 352 known and putative autoAgs are COVID-altered proteins

To find out which autoAgs may be involved in COVID-19, we compared the DS-affinity autoantigenome with proteins and genes that are up- or down-regulated in SARS-CoV-2 infection (Coronascape database comparison, Supplemental Table 1) (28–48). Remarkably, 352 (86.3%) of the 408 DS-affinity proteins have been found to be altered (up- and/or down-regulated at protein and/or mRNA levels) in COVID-19 patients or SARS-CoV-2 infected cells (Table 1). Of these, 260 are reported as up-regulated and 303 as down-regulated (including 211 that are both up- and down-regulated). The numbers are not conflicting, because the COVID data were generated by multiple proteomic and transcriptomic methods and different cells and tissues. A protein may not be overexpressed even when its mRNA is up-regulated, and a protein/gene may be up-regulated in one tissue or patient but down-regulated in another tissue or patient. A protein is considered altered if it is up- or down-regulated at the protein or RNA level and, in relation to SARS-CoV-2 infection, it is considered a COVID-altered protein.

**Fig. 2.**
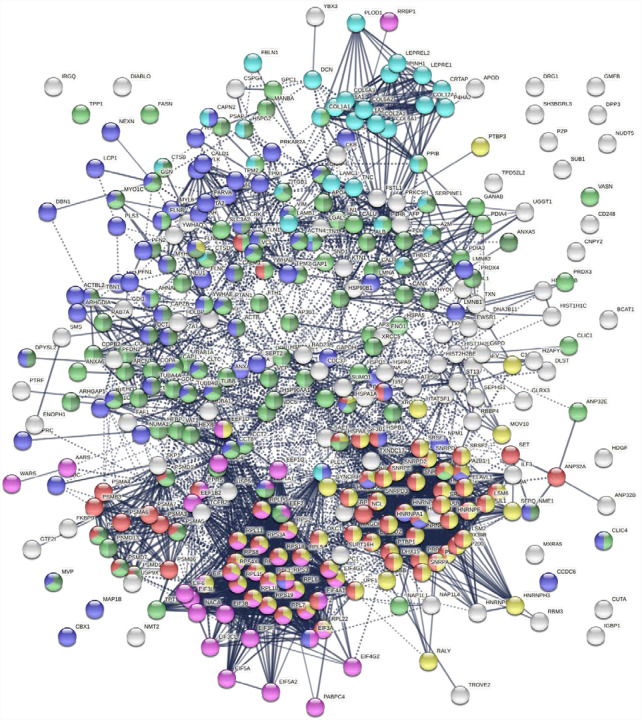
Network of 352 autoantigenome proteins that are altered in SARS-CoV-2 infected cells or patients. Connecting lines represent interactions with high confidence. Colored proteins are involved in metabolism of RNA (77 proteins, red), mRNA metabolic process (69 proteins, gold), translation (43 proteins, pink), vesicles (99 proteins, light green) and vesicle-mediated transport (84 proteins, dark green), cytoskeleton (84 proteins, blue), and extracellular matrix organization (32 proteins, aqua).

Protein-interaction analysis revealed that 352 COVID-altered proteins form a highly connected network, exhibiting 6,286 interactions (vs. 2,451 expected; PPI enrichment p-value <1.0E-6) (Fig. 2). Based on cellular component analysis, the altered proteins can be located to intracellular organelles (323 proteins), nucleus (199 proteins), endomembrane system (143 proteins), vesicles (99 proteins), ribonucleoprotein complex (87 proteins), cytoskeleton (84 proteins), ER (72 proteins), and cell projections (52 proteins). Organelles with significant numbers of component proteins identified include the melanosome (30/105 proteins in melanosome), proteasome (16/64), polysome (13/66), spliceosome (34/187), ficolin-1-rich granule lumen (22/125), azurophil granules (17/155), and myelin sheath (26/157).

Similarly, the group of 260 up-regulated proteins is highly connected (3,747 interactions vs. 1,424 expected) with significant enrichment in proteins associated with RNA and mRNA metabolism, translation, vesicles and vesicle-mediated transport, and regulation of cell death (Fig. 3A). The group of 303 down-regulated proteins is also highly connected (4,860 interactions vs. 1,907 expected), and these proteins are significantly related to RNA metabolism, translation, vesicles, cytoskeleton, and extracellular matrix (Fig. 3B).

### Pathways and processes affected by COVID-altered proteins

Network enrichment analysis by Metascape revealed that the 352 COVID-altered proteins are most significantly enriched in RNA metabolism, axon guidance, and translation (Table 2). Many processes, e.g., regulated exocytosis, wound healing, supramolecular fiber organization, smooth muscle contraction, and platelet degranulation are significantly affected by COVID-altered proteins regardless of whether they are up- or down-regulated. The up-regulated proteins are more related to axon guidance and interleukin signaling, whereas down-regulated proteins are more related to cellular response to stress and apoptosis.

### COVID-altered autoAgs are strongly related to the nervous system

COVID-19 patients frequently report neurological problems, such as loss of smell and taste, dizziness, headache, and stroke. While most symptoms are transient, some recovered patients are haunted by lingering neurological and psychological problems long after the viral infection. The underlying cause of transient and long-lasting neurological effects of COVID-19 has been puzzling. Analysis of COVID-altered proteins revealed a strong link to the nervous system. Of the 352 COVID-altered proteins, at least 150 are related to the nervous system (Fig. 4A). More than 60 proteins are related to axon guidance based on ontology analyses (Table 2 and Fig. 4A). In addition, there are 39 proteins related to neuron projection, 26 proteins related to myelin sheath, 25 proteins related to axon growth cone (52), 16 proteins related to neuronal cell body, 4 proteins related to cerebellar Purkinje cell layer, 3 proteins related to peripheral nervous system axon regeneration, and 2 proteins related to radial glial scaffolds. In particular, we found that 23 COVID-altered proteins are related to the olfactory bulb (53), which may explain the loss of smell in many COVID-19 patients.

Most of these proteins are known autoAgs, e.g., ACTB, CANX, A2M, APOA1, CAPZA1, DPYSL2, FLNA, GDI2, LGALS1, MSN, PDIA3, PFN2, TNC, UCHL1, VCP, and VCL (see autoAg references in Table 1). Some yet-to-be-confirmed autoAgs with direct relation to the nerve system, e.g., NES (expressed mostly in nerve cells) and APOD (expressed by oligodendrocytes), warrant further investigation.

The COVID-altered proteins are also associated with a number of neurological diseases (Fig. 4B). By comparing our data with published proteomes, 23 proteins were similarly found in neuronal infection by Japanese encephalitis virus (54), 21 proteins in neuroblastoma (55), 22 proteins in glioblastoma (56), 26 proteins in neurodegeneration in Down syndrome (57), 22 proteins in Alzheimer disease hippocampus (58), 24 proteins in schizophrenia (59), 17 proteins in cerebral ischemia (60), and 17 proteins in Parkinson disease (61).

Coronavirus-induced demyelination has been reported in a mouse model of multiple sclerosis (62), which may explain our identification of 26 altered proteins related to the myelin sheath in SARS-CoV-2 infection. In a mouse brain injury model, DS appears to play an important role in glial scar formation and regeneration of dopaminergic axons (63). Alterations of white matter DS and extracellular matrix are specific, dynamic, and widespread in multiple sclerosis patients (64). DS has recently been reported to promote neuronal differentiation in mouse and human neuronal stem cells (65). Given the various functional roles of DS, our identification of a large number of known and putative autoAgs with DS affinity related to the nervous system is a compelling finding.

### COVID-altered autoAgs are related to cell death, wound healing, and blood coagulation

SARS-CoV-2 infection causes host cell death and leads to tissue injury. Wound healing, cellular response to stress, and apoptosis are among the most significant processes related to COVID-altered proteins (Table 2 and Fig. 5A). For example, we identified 66 proteins related to regulation of cell death and 23 related to regulation of apoptotic signaling pathways. DS binds to apoptotic cells and autoAgs released from dying cells, which has led to our previous identification of hundreds of autoAgs (13–16, 18). Upon tissue injury, DS biosynthesis is ramped up by fibroblasts and epithelial and endothelial cells (7–9). After tissue injury, DS assists fibroblast migration into the wound to facilitate granulation tissue formation and wound healing (11). DS, similar to heparin, is also an important anticoagulant that inhibits clot formation via interaction with antithrombin and heparin cofactor II (66). Given these biological roles of DS, it is consistent that a large number of COVID-altered proteins related to cell death and tissue injury are identified by DS-affinity.

Blood coagulation and thrombosis are frequent complications of COVID-19. Platelet degranulation is found to be significantly associated with at least 18 altered proteins (Table 2 and Fig. 5A). COVID-altered proteins are related to blood coagulation, platelet activation, platelet alpha granules, fibrinogen binding, fibrinogen complex, platelet plug formation, von Willebrand factor A-like domain superfamily, and platelet-derived growth factor binding. Collagens, which support platelet adhesion and activation, and collagen biosynthesis and modifying enzymes are also among the COVID-altered proteins, e.g., collagen type VI trimer and type I trimer (Fig. 5A). The majority of these altered proteins are known autoAgs, e.g., ALB, ANXA5, C1QBP, CALM1, CAPZB, COL1A1, COL1A2, COL6A1, FBLN1, FN1, PLEC, PPIB, THBS1, TLN1, TUBA4A, and YWHAZ (see autoAg references in Table 1). Some are unknown and await further investigation, e.g., AP3B1, CRK, CTSB, EHD2, PLOD1, PSAP, and PARKAR2A.

### Supramolecular fibril alteration offers clues to muscle dysfunction and fibrosis

Over 50 supramolecular filament proteins are identified by DS-affinity from HFL1 cells. Remarkably, nearly all (except for one) are found to be altered in SARS-CoV-2 infection, and the majority have already been reported as autoAgs (Table 1). They include various isoforms of actin, actinin, collagen, filamin, fibronectin, fibulin, dynactin, dynein, lamin, myosin, nestin, nexilin, profilin, plectin, plastin, proteoglycan, septin, spectrin, talin, tropomyosin, tubulin, vinculin, and vimentin (Table 1, Fig. 5B). These proteins are major components of the extracellular matrix, basement membrane, cell cytoskeleton, cytoskeletal motors, muscle filaments, and contractile motors of muscle cells.

A significant number of COVID-altered proteins are related. Emerin complex and smooth muscle contraction are among the top enriched biological processes of COVID-altered proteins (Table 2, Fig. 5B). Emerin is highly expressed in cardiac and skeletal muscle, and emerin mutations cause X-linked recessive Emery-Dreifuss muscular dystrophy, cardiac conduction abnormalities, and dilated cardiomyopathy. Smooth muscle resides primarily in the walls of hollow organs where it performs involuntary movements, e.g., respiratory tract, blood vessels, gastrointestinal tract, and renal glomeruli. In addition, we identified proteins with significant association to myofibrils (the contractile elements of skeletal and cardiac muscle; 23 proteins) (Fig. 5B), stress fiber (a contractile actin filament bundle that consists of short actin filaments with alternating polarity: MYH9, MYLK, FLNB, TPM1, TPM2, TPM3, TPM4, ACTN1, ACTN4), muscle filament sliding (the sliding of actin thick filaments and myosin thick filaments past each other in muscle contraction), Z disk (plate-like region of a muscle sarcomere to which the plus ends of actin filaments are attached), intercalated disc (a cell-cell junction complex at which myofibrils terminate in cardiomyocytes, mediates mechanical and electrochemical integration between individual cardiomyocytes), and negative regulation of smooth muscle cell-matrix adhesion (2 proteins; SERPINE1, APOD).

Pulmonary fibrosis is prominent in COVID-19 and contributes to lethality in some cases (67, 68). Fibrosis, or fibrotic scarring, is pathological wound healing in which excessive extracellular matrix components are produced by fibroblasts and accumulate in the wounded area. Histopathological examination of COVID-19 patients found highly heterogenous injury patterns reminiscent of exacerbation of interstitial lung disease, including interstitial thickening, fibroblast activation, and deposition of collagen fibrils (22). We identified a significant number of COVID-altered proteins that are associated with collagen bundles and collagen biosynthesis and modifying enzymes (16 proteins), extracellular matrix organization (33 proteins), supramolecular fibers, and amyloid formation offering functional links to fibrosis (Fig. 5B).

### Potential autoAgs in COVID-19 patients and a connection to the melanosome

**Fig. 6.**
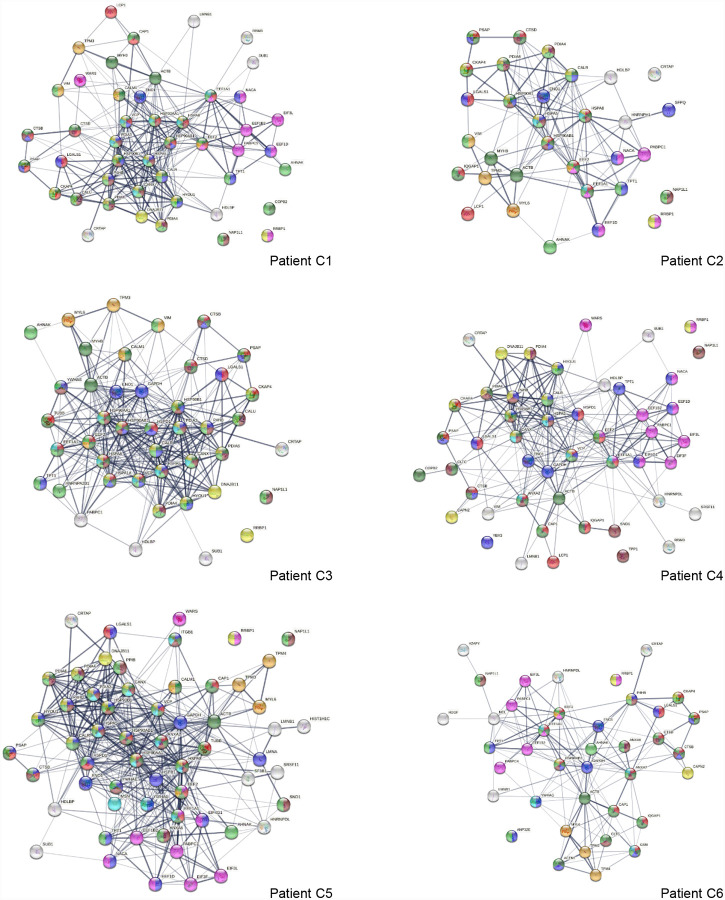
Interaction network of altered proteins in 6 COVID-19 patients. Colored proteins are associated with leukocyte activation involved in immune response (red), vesicles (light green) and vesicle-mediated transport (dark green), protein processing in the ER (yellow), regulation of cell death (blue), translation (pink), melanosome (brown), myelin sheath (aqua), and muscle contraction (amber).

To find out how altered proteins may differ in patients, we compared our putative autoantigenome to published single-cell RNA sequencing data of 6 patients hospitalized for COVID-19 (28, 34) and identified 32–59 putative autoAgs per patient (Fig. 6). Interestingly, while identified from different patients, the altered proteins/genes identified share involvement of leukocyte activation, vesicles and vesicle transport, protein processing in the ER (including antigen processing and presentation), regulation of cell death, translation, muscle contraction, myelin sheath, and curiously, the melanosome (Fig. 6). The estrogen signaling pathway and thyroid hormone synthesis are found to be associated with altered proteins in some patients. Patient C2 has 5 altered proteins related to neuron differentiation regulation, and patient C4 has 6 altered proteins related to neuron death.

Eleven altered proteins were identified in all 6 patients, including known autoAgs (ACTB, EEF1A1, EEF2, ENO1, LGALS1, PABPC1) and unknown ones (CRTAP, NAP1L1, PSAP, RRBP1, TPT1) (Table 1). AHNAK (neuroblast differentiation-associated protein, a known autoAg in lupus) was identified in 5 patients. Overall, a majority of the altered proteins identified from the 6 COVID patients are known autoAgs, e.g., CALM1, CALR, CALU, CANX, DNAJB11, HDGF, HSPA5 (BiP), IQGAP1, LCP1, LMNB1, MYH9, NACA, P4HB, SFPQ, PDIA3, TPM3, TUBB, VCP, VIM, WARS, and YB3 (Table 1). Unknown or putative autoAgs include CAP1, CTSB, HDLBP, HYOU1, SND1, and SUB1.

We initially identified 30 DS-affinity proteins from HFL1 cells related to the melanosome, and, intriguingly, all of these are also COVID-altered proteins (Fig. 5B). Based on STRING GO analysis, the melanosome is the most significant cellular component related to altered proteins in all 6 patients (with false discovery rates ranging from 1.52E-8 to 1.11E-23). In HIV infection, melanosome production is stimulated in some patients and leads to an increase in pigmented lesions (69). However, melanosome involvement in COVID-19 is not known. Two Wuhan doctors in intensive care for COVID temporally turned dark, although the cause was thought to be a drug reaction. A COVID patient has been reported with acute flaccid tetraparesis and maculopapular pigmented plaques on the limbs (70). In mice, coronavirus induces an acute and long-lasting retinal disease, with initial retinal vasculitis followed by retinal degeneration that is associated with retinal autoantibodies and retinal pigment epithelium autoantibodies (71).

### Association between autoimmunity and virus infections

We identified COVID-altered proteins with DS-affinity that are involved in the host response to various aspects of viral infection and that possess a high propensity to become autoAgs. For example, viral RNA metabolism, translation, vesicles, and vesicle transport contribute a large number of known and putative autoAgs. In addition, viral processes, particularly symbiont processes and interspecies interactions between host and viruses, contribute significantly to altered proteins (Fig. 7A). For example, among altered proteins related to response to viral processes, HSPA8, DDB1, RAD23A, PABPC1, PPIB, P4HB, LGALS1, GSN, and ILF3 are known autoAgs (Table 1).

In particular, COVID-altered cytoskeletal filament proteins shed light on viral trafficking in host cells. SARS-CoV-2 infection induces profound remodeling of the cytoskeleton, and replicating viral vesicles are surrounded by a network of intermediate filaments (72). The cytoskeletal network appears to facilitate coronavirus transport and expulsion, with thickening actin filaments providing the bending force to extrude viral vesicles (73). We identified 84 altered proteins related to the cytoskeleton and 84 altered proteins related to vesicle-mediated transport (Fig. 2). These altered proteins are implicated in various processes, including cytoskeleton-dependent intracellular transport, actin fiber-based movement, actin-mediated cell contraction, microtubule-dependent trafficking from the Golgi to the plasma membrane, and transport along microtubules.

Many positive-strand RNA viruses (including SARS-CoV-2, Enterovirus, Hepatitis C virus, Norovirus, and Poliovirus) hijack a common group of nuclear factors to support the biosynthetic functions required for viral replication and propagation (74). 20 of these hijacked nuclear proteins are identified by DS-affinity in our study (Fig. 7). In addition, altered proteins are found in other viral infections, including porcine reproductive and respiratory syndrome virus (75), H5N1 avian influenza viruses (76, 77), Japanese encephalitis virus (54), Rift Valley fever virus (78), Hepatitis B virus (79), HIV (80–82), Herpes Simplex virus (83), and Epstein-Barr virus infection (Fig. 7B and STRING ontology analysis).

### Autoimmunity concerns for mRNA vaccines

Our study identified a large number of known and putative autoAgs that are related to mRNA metabolism, translation, vesicles, and vesicle trafficking (Figs. 1–2). This finding begs us to wonder whether mRNA vaccines may induce unintended autoimmune consequences in the long term. mRNA vaccines are essentially synthetic viral vesicles. To induce protective immunity, mRNA vaccine vesicles will need to be transported into cells where they hijack the host cell machinery to produce a viral protein antigen, whereupon the antigen will be processed and presented by MHC molecules to induce B and T cell responses.

mRNA translation requires ribosomes, translation initiation factors, aminoacyl-tRNA synthetases, and elongation factors. We identified 18 ribosomal proteins by DS-affinity, all of which are altered in SARS-CoV-2 infection and 9 of which are known autoAgs (see references in Table 1). We also identified 15 eukaryotic translation initiation factor proteins, with 12 of them being COVID-altered and 4 being known autoAgs (Table 1). Six elongation factor proteins (5 subunits of EEF1 complex, EEF2) were identified by DS-affinity, of which all 6 are COVID-altered and 3 are known autoAgs (Table 1). Six tRNA synthetases were identified, with 5 being known autoAgs and 3 (AARS, EPRS, WARS) COVID-altered (Table 1). Autoantibodies to AARS are associated with interstitial lung disease and myositis (84, 85). EPRS appears to regulate pro-fibrotic protein synthesis during cardiac fibrosis (86). Gene mutations of WARS cause an autosomal dominant neurologic disorder characterized by slowly progressive distal muscle weakness and atrophy affecting both the lower and upper limbs (87, 88).

Once synthesized, the exogenous protein antigens are degraded by proteasomes, and the resulting peptides are transported into the ER where they are loaded onto MHC molecules by peptide loading complexes for presentation to T cells. In relation to these steps, 15 proteasome subunits were identified by DS-affinity, with 12 being COVID-altered and 7 being known autoAgs (Table 1). Nine proteins related to antigen processing and presentation are found to be altered in the 6 COVID-19 patients analyzed in this study, including HSPA1A, HSPA8, HSP90AA1, HSPAB1, HSPA5, PDIA3, CANX, CALR, and CTSB, with 7 being known autoAgs (Fig. 5 and Table 1).

In addition, among the 352 COVID-altered proteins identified in this study, 69 proteins are associated with mRNA metabolism (Fig. 2). Many of these proteins may be irrelevant to non-replicating mRNA molecules in mRNA vaccines, however, some are likely needed in processes such as 3’ end processing, deadenylation, and nonsense-mediated decay. For example, we identified poly(A) tail binding proteins PABPC1 and PABPC4 as COVID-altered proteins, both of which have been reported as autoAgs (Table 1).

mRNA vaccines are synthetic vesicles. This study identified 99 altered proteins associated with vesicles and 84 proteins associated with vesicle-mediated transport (Figs. 1, 2, 5). Although it is not clear which host molecules are involved in extra- and intracellular transport and uptake of mRNA vaccine vesicles, some of the vesicle-related proteins identified as DS-affinity proteins may be involved, e.g., proteins of receptor-mediated endocytosis (APOA1, CALR, CANX, CAP1, CLTC, HSP90AA1, HSP90B1, HSPG2, ITGB1, YWHAH) or phagocytosis (ACTB, CRK, GSN, HSP90AA1, HSP90AB1, MYH9, MYO1C, PDIA6, RAB7A, THBS1, TXNDC5).

Overall, a significant number of autoAgs related to different steps of mRNA vaccine action were identified in this study; however, our findings do not mean that these autoAgs will lead to aberrant autoimmune reactions as a result of mRNA vaccination. The development of autoimmune diseases or autoimmunity-related diseases entails a complex cascade of molecular and cellular interactions. Long-term monitoring of autoimmune adverse effects will be needed.

## Conclusion

This study identifies an autoantigenome of 408 proteins from human fetal lung fibroblast HFL1 cells by DS-affinity and protein sequencing, of which at least 231 proteins are confirmed autoAgs. Of these, 352 (86.3%) are found to be altered in SARS-CoV-2 infection when compared to published data, with at least 210 COVID-altered proteins being known autoAgs. The altered proteins are significantly enriched in a number of pathways and processes and are closely connected to various disease manifestations of COVID-19, particularly neurological problems, fibrosis, muscle dysfunction, and thrombosis.

Viral infections cause significant perturbations of normal cellular and tissue component molecules in the host, leading to cell death and tissue injury. Autoantigens resulting from molecular alterations may result directly from the injury or indirectly from responses to the injury. As a stress response, DS biosynthesis may be ramped up to facilitate wound healing and dead cell clearance. DS associates with autoAgs and stimulates autoreactive B cells and autoantibody production. Specific autoantibodies that are initially induced in response to a certain injury site may circulate and attack secondary sites where the autoAgs are also expressed, leading to a complex array of local and systemic autoimmune diseases.

This study illustrates a strong connection between viral infection and autoimmunity. The COVID-19 autoantigenome provides a detailed molecular map for investigating the diverse spectrum of autoimmune sequelae caused by the pandemic. The autoantigen atlas we are establishing may also serve as a detailed molecular reference for monitoring possible autoimmune reactions to mRNA vaccines and other viral infections.

## Materials and Methods

### HFL1 cell culture

The HFL1 cell line was obtained from the ATCC (Manassas, VA, USA) and cultured in Eagle’s Minimum Essential Medium supplemented with 10% fetal bovine serum (Thermo Fisher) and a penicillin-streptomycin-glutamine mixture (Thermo Fisher) at 37 °C.

### Protein extraction

About 100 million cells were harvested and suspended in 10 ml of 50 mM phosphate buffer (pH 7.4) containing the Roche Complete Mini protease inhibitor cocktail. Cells were homogenized on ice with a microprobe sonicator until the turbid mixture became nearly clear with no visible cells left. The homogenate was centrifuged at 10,000 g at 4 °C for 20 min, and the supernatant was collected as the total protein extract. Protein concentration was measured with the RC DC protein assay (Bio-Rad).

### DS-Sepharose resin preparation

20 ml of EAH Sepharose 4B resins (GE Healthcare Life Sciences) were washed with distilled water three times and mixed with 100 mg of DS (Sigma-Aldrich) in 10 ml of 0.1 M MES buffer, pH 5.0. 500 mg of N-(3-dimethylaminopropyl)-N’-ethylcarbodiimide hydrochloride (Sigma-Aldrich) powder was added to the mixture. The reaction proceeded by end-over-end rotation at 25 °C for 16 h. After coupling, resins were washed with water and equilibrated first with a low-pH buffer (0.1 M acetate, 0.5 M NaCl, pH 5.0) and then with a high-pH buffer (0.1 M Tris, 0.5 M NaCl, pH 8.0).

### DS-affinity fractionation

The total proteins extracted from HFL1 cells were fractionated on DS-Sepharose columns with a BioLogic Duo-Flow system (Bio-Rad). About 40 mg of proteins in 40 ml of 10 mM phosphate buffer (pH 7.4; buffer A) were loaded onto the column at a rate of 1 ml/min. Unbound proteins were washed off with 60 ml of buffer A, and weakly bound proteins were eluted with 40 ml of 0.2 M NaCl in buffer A. DS-binding proteins were eluted with sequential 40-ml step gradients of 0.5 M and 1.0 M NaCl in buffer A. Fractions were desalted and concentrated to 0.5 ml with 5-kDa cut-off Vivaspin centrifugal filters (Sartorius). Fractionated proteins were separated by 1-D SDS-PAGE in 4–12% Bis-Tris gels, and the gel lanes corresponding to 1.0 M or 0.5 M NaCl elutions were divided into two or three sections for sequencing.

### Mass spectrometry sequencing

Protein sequencing was performed at the Taplin Biological Mass Spectrometry Facility at Harvard Medical School. Proteins in gels were digested with sequencing-grade trypsin (Promega) at 4 °C for 45 min. Tryptic peptides were separated on a nano-scale C_18_ HPLC capillary column and analyzed in an LTQ linear ion-trap mass spectrometer (Thermo Fisher). Peptide sequences and protein identities were assigned by matching the measured fragmentation pattern with proteins or translated nucleotide databases using Sequest. All data were manually inspected. Only proteins with ≥2 peptide matches were considered positively identified.

### COVID data comparison with Coronascape

DS-affinity proteins were compared with currently available proteomic and transcriptomic data from SARS-CoV-2 infection compiled in the Coronascape database (as of 12/14/2020) (28–48). These data had been obtained with proteomics, phosphoproteomics, interactome, ubiquitome, and RNA-seq techniques. Up- and down-regulated proteins or genes were identified by comparing COVID-19 patients vs. healthy controls and cells infected vs. uninfected by SARS-CoV-2. Similarity searches were conducted between our data and the Coronascape database to identify DS-affinity proteins (or their corresponding genes) that are up- and/or down-regulated in the viral infection.

### Pathway and process enrichment analysis

Pathways and processes enriched in the putative autoantigenome were analyzed with Metascape (28). The analysis was performed with various ontology sources, including KEGG Pathway, GO Biological Process, Reactome Gene Sets, Canonical Pathways, CORUM, TRRUST, and DiGenBase. All genes in the genome were used as the enrichment background. Terms with a p-value <0.01, a minimum count of 3, and an enrichment factor (ratio between the observed counts and the counts expected by chance) >1.5 were collected and grouped into clusters based on their membership similarities. The most statistically significant term within a cluster was chosen to represent the cluster. Pathway hierarchical clustering was obtained with ShinyGo (89).

### Protein-protein interaction network analysis

Protein-protein interactions among collections of DS-affinity proteins were analyzed by STRING (51), including both direct physical interaction and indirect functional associations. Interactions are derived from genomic context predictions, high-throughput lab experiments, co-expression, automated text mining, and previous knowledge in databases. Each interaction is annotated with a confidence score from 0 to 1, with 1 being the highest, indicating the likelihood of an interaction to be true. Only interactions with high confidence (a minimum score of 0.7) are shown in the figures.

### Literature text mining

Literature searches in Pubmed were performed for every DS-affinity protein identified in this study. Search keywords included the protein name, its gene symbol, alternative names and symbols, and the MeSH keyword “autoantibodies”. Only proteins with their specific autoantibodies reported in PubMed-listed journal articles were considered “confirmed” autoAgs in this study.

## Supplementary Material

Supplement 1

## Figures and Tables

**Fig. 3A.**
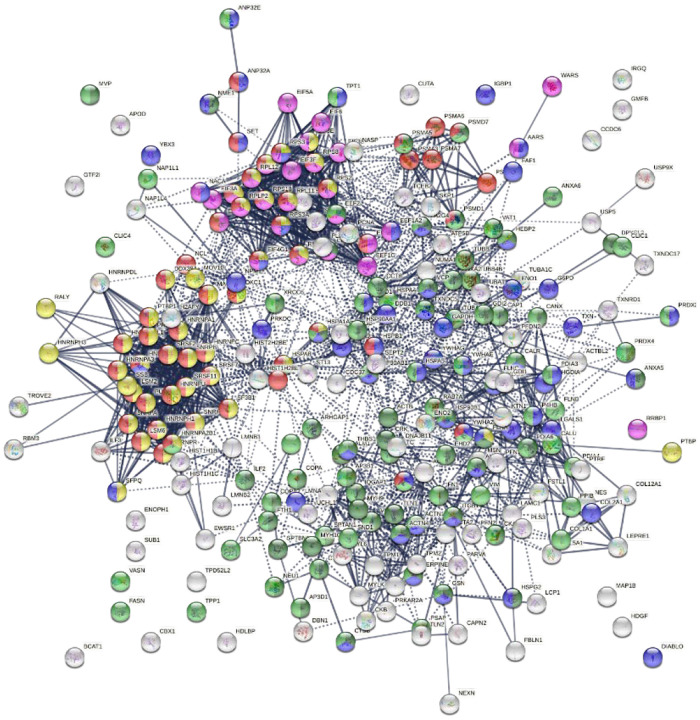
Interaction network of 260 up-regulated proteins in SARS-CoV-2 infected cells or patients. Connecting lines represent interactions with high confidence (minimum interaction score of 0.7). Colored proteins are involved in metabolism of RNA (54 proteins, red), translation (28 proteins, pink), vesicles (82 proteins, light green) and vesicle-mediated transport (67 proteins, dark green), regulation of cell death (61 proteins, blue), and mRNA metabolic process (46 proteins, gold).

**Fig. 3B.**
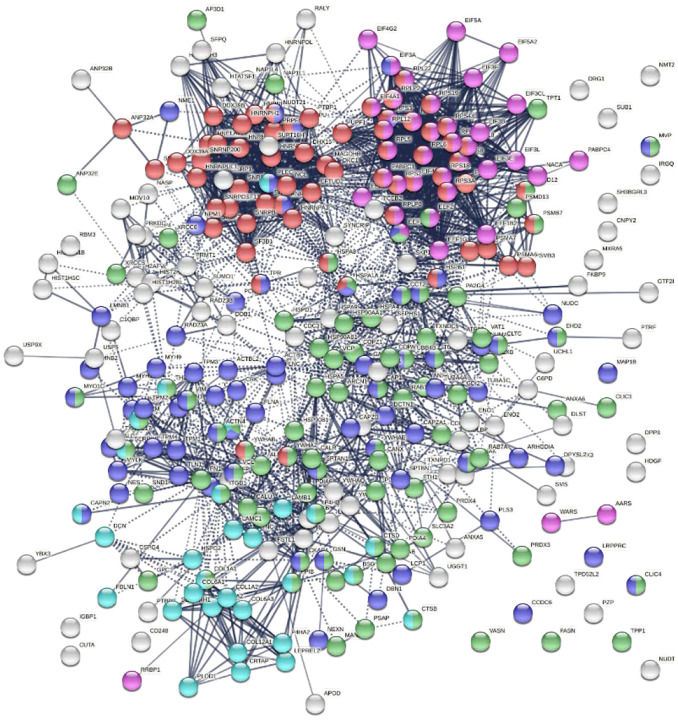
Interaction network of 303 down-regulated proteins in SARS-Cov-2 infected cells and patients. Connecting lines represent interactions with high confidence. Marked proteins are involved in RNA metabolism (64 proteins), translation (39 proteins, pink), vesicles (88 proteins, green), cytoskeleton (73 proteins, blue), and extracellular matrix organization (29 proteins, aqua).

**Fig. 4A.**
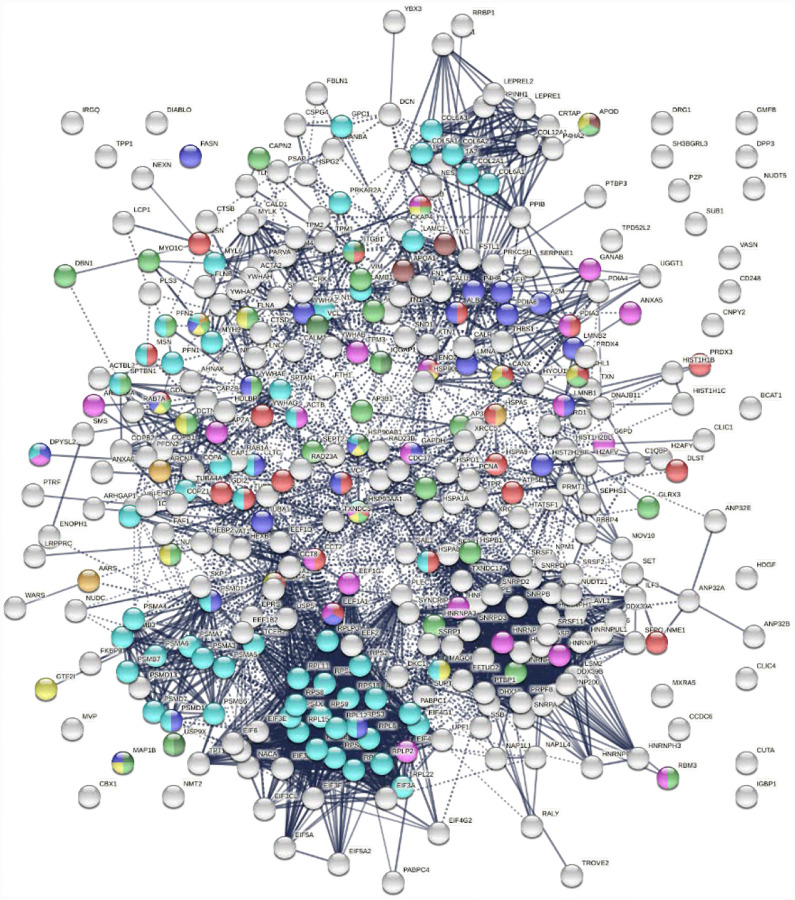
Nervous system-related proteins among COVID-altered proteins. Colored proteins are involved in axon guidance (62 proteins, aqua), axon growth cone (25 proteins, blue), myelin sheath (26 proteins, red), neuron projection (32 proteins, green) and neuron projection extension (7 proteins, dark green), neuronal cell body (16 proteins, gold), peripheral nervous system axon regeneration (3 proteins, brown), cerebellar Purkinje cell layer development (4 proteins, amber), and olfactory bulb (23 proteins, pink).

**Fig. 4B.**
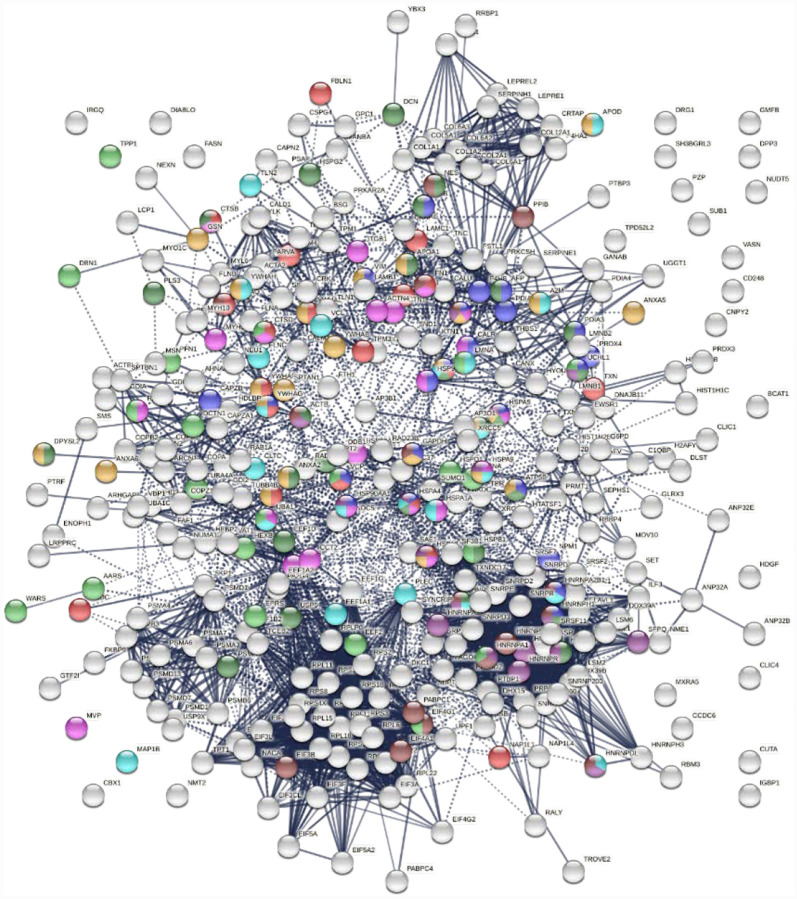
Neurological disease-related proteins among proteins altered in COVID. Colored are proteins found in neuronal infection with Japanese encephalitis virus (23 proteins, blue), neuroblastoma (21 proteins, red), glioblastoma (22 proteins, pink), neurodegeneration in Down syndrome (26 proteins, dark green), Alzheimer disease (22 proteins, aqua), schizophrenia (24 proteins, amber), cerebral ischemia induced neurodegenerative diseases (17 proteins, dark purple), Parkinson disease (17 proteins, brown), and neurodegeneration (21 proteins, green).

**Fig. 5A.**
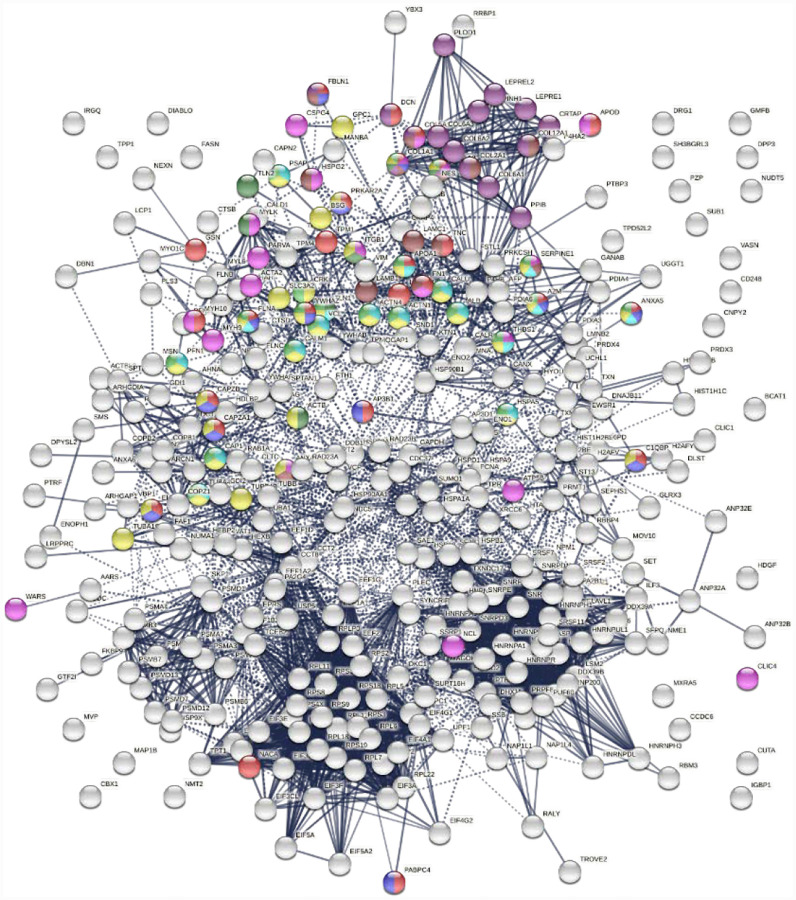
Relation of COVID-altered proteins to wound healing and hemostasis. Response to wounding (25 proteins, red), blood vessel development (20 proteins, pink), blood coagulation (14 proteins, blue), collagen-containing extracellular matrix (13 proteins, brown), collagen biosynthesis and modifying enzymes (16 proteins, dark purple), platelet activation (3 proteins, dark green) and platelet activation signaling and aggregation (22 proteins, green), platelet degranulation (18 proteins, aqua), and hemostasis (35 proteins, gold).

**Fig. 5B.**
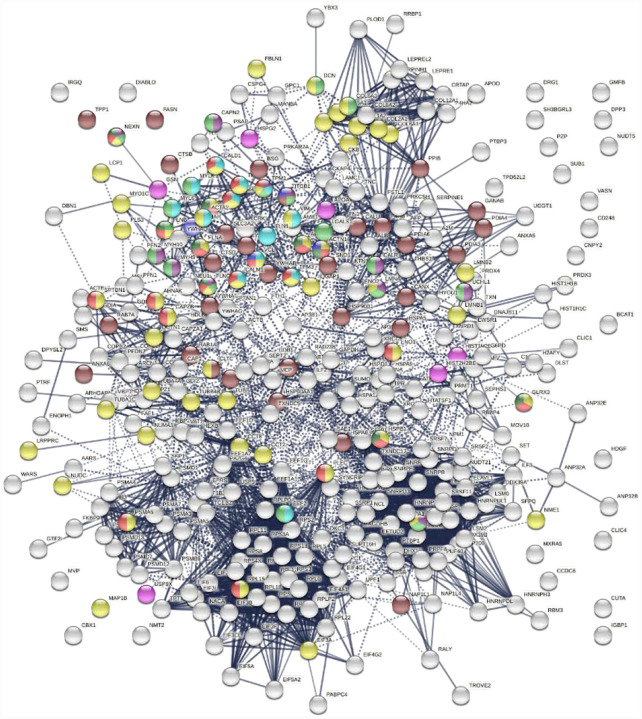
Other significantly enriched groups among altered proteins. Supramolecular fiber (56 proteins, amber), melanosome (30 proteins, brown), striated muscle cell differentiation (11 proteins, purple), myofibril (23 proteins, red), muscle structure development (18 proteins, green), muscle contraction (13 proteins, aqua), Z disk (9 proteins, dark green), intercalated disk (4 proteins, blue), and amyloid fiber formation (6 proteins, pink).

**Fig. 7A.**
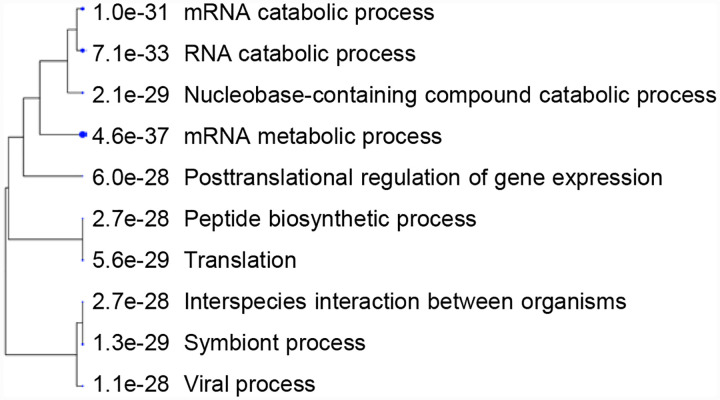
Hierarchical clustering of top 10 pathways involving COVID-altered proteins. Analysis based on hypergeometric distribution followed by FDR correction.

**Fig. 7B.**
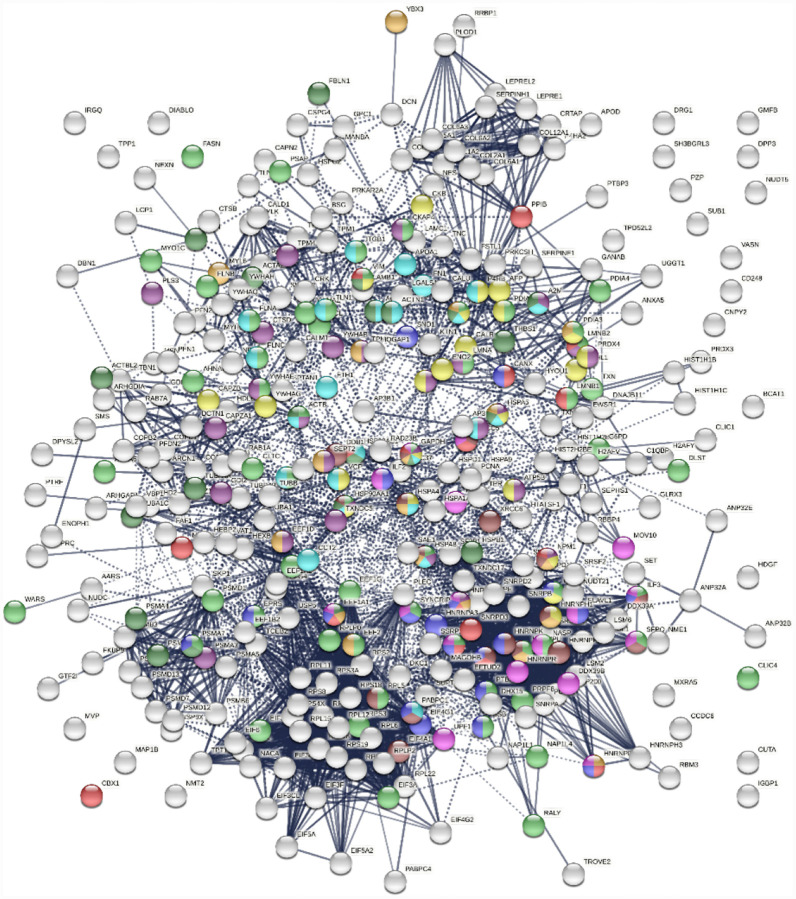
COVID-altered host proteins with DS-affinity found in various viral infections. Porcine reproductive and respiratory syndrome (56 proteins, green), H5N1 avian influenza virus (27 proteins, dark purple), Japanese encephalitis virus (23 proteins, gold), Rift Valley fever virus (24 proteins, aqua), Hepatitis B virus (22 proteins, dark green), HIV (identified in different studies, 18 amber, 18 brown, 18 red and 17 pink), and shared among positive-sense RNA viruses (20 proteins, blue).

**Table 1.** DS-affinity enriched autoantigenome from human HFL1 cells

# Pep.	Gene	Protein	COVID		DS affinity		Ref.
			Up	Down	1.0 M	0.5 M	
5	A2M	Alpha-2-macroglobulin		D		+	(1)
5	AARS	Alanyl-tRNA synthetase, cytoplasmic	U	D		+	(2)
10	ACTA2	Actin, aortic smooth muscle	U	D		+	(3)
8	ACTB	Actin, cytoplasmic	U	D		+	(4)
6	ACTBL2	Beta-actin-like protein	U	D		+	
17	ACTN1	Alpha-actinin-1	U	D		+	(5)
6	ACTN4	Alpha-actinin-4	U	D		+	(3)
3	AFP	Alpha-fetoprotein		D		+	(6)
5	AHNAK	Neuroblast differentiation-associated protein	U	D		+	(7)
10	ALB	Putative uncharacterized protein albumin	U	D		+	(8)
3	ALPP	Alkaline phosphatase, placental type precursor			+		(9)
6	ANP32A	Acidic leucine-rich nuclear phosphoprotein 32 member A	U	D	+		
11	ANP32B	Acidic nuclear phosphoprotein 32 family member B		D	+		
3	ANP32C	Acidic nuclear phosphoprotein 32 family member C			+		
3	ANP32E	Acidic nuclear phosphoprotein 32 family member E	U	D	+		
2	ANXA2	Annexin A2	U	D	+		(10)
7	ANXA2P2	Putative annexin A2-like protein, ANX2L2, LPC2B	U	D		+	
7	ANXA5	Annexin A5	U	D		+	(11)
33	ANXA6	Annexin VI	U	D		+	(12)
2	AP1B1	AP-1 complex subunit beta-1				+	
2	AP3B1	AP-3 complex subunit beta-1	U		+		
2	AP3B2	AP-3 complex subunit beta-2			+		(13)
3	AP3D1	AP-3 complex subunit delta-1	U	D	+		
3	APOA1	Apolipoprotein A-I		D		+	(14)
2	APOD	Apolipoprotein D	U	D		+	
2	ARCN1	Coatomer delta, Archain vesicle transport protein 1		D		+	
4	ARF1	ADP-ribosylation factor				+	
2	ARHGAP1	Rho-GTPase-activating protein	U			+	
4	ARHGDIA	Rho GDP-dissociation inhibitor 1	U	D		+	
9	ATP5B	ATP synthase subunit beta, ATP5F1B	U	D		+	(15)
3	BCAT1	Branched chain amino acid aminotransferase	U			+	
2	BCCIP	BRCA2 and CDKN1A-interacting protein				+	
2	BGN	Biglycan			+		(16)
2	BSG	Basigin, CD147		D	+		(17)
2	BZW2	Basic leucine zipper and W2 domains 2				+	
7	C1QBP	Complement C1q-binding protein		D	+		(18)
7	CALD1	Caldesmon		D		+	
8	CALM1	CALM3; CALM2 Calmodulin	U	D		+	(19)
16	CALR	Calreticulin	U	D		+	(20)
2	CALU	Calumenin	U	D		+	(21)
3	CANX	Calnexin	U	D	+		(22)
9	CAP1	Adenylyl cyclase-associated protein	U	D		+	
7	CAPN1	Calpain-1 catalytic subunit				+	
5	CAPN2	Calpain-2 catalytic subunit	U	D		+	(19)
3	CAPNS1	Calpain small subunit				+	
2	CAPZA1	F-actin-capping protein subunit alpha-1		D	+		(23)
3	CAPZB	F-actin-capping protein subunit beta		D		+	(24)
8	CAVIN1	Caveolae-associated protein 1, PTRF	U	D		+	
3	CBX1	Chromobox protein homolog	U			+	(25)
3	CCDC6	Coiled-coil domain-containing protein	U	D		+	(26)
3	CCT2	T-complex protein 1 subunit beta		D		+	
3	CCT8	T-complex protein 1 subunit theta	U	D		+	(27)
4	CD248	Endosialin		D		+	
5	CDC37	Hsp90 co-chaperone Cdc37	U	D		+	
4	CKAP4	Cytoskeleton-associated protein 4, P63	U	D	+		(28)
8	CKB	Creatine kinase B-type	U	D		+	(29)
7	CLIC1	Chloride intracellular channel protein	U	D		+	
2	CLIC4	Chloride intracellular channel protein	U	D		+	
14	CLTC	Clathrin heavy chain 1	U	D	+		(30)
3	CLTCL1	Clathrin heavy chain 2			+		
3	CNPY2	Protein canopy homolog		D		+	
13	COL12A1	Collagen type XII alpha-1 chain	U	D		+	
45	COL1A1	Collagen type I alpha-1 chain	U	D		+	(31)
37	COL1A2	Collagen type I alpha-2 chain		D		+	(32)
2	COL2A1	Collagen type II alpha-1 chain	U			+	(33)
12	COL3A1	Collagen type III alpha-1 chain				+	(34)
3	COL5A1	Collagen type V alpha 1	U			+	(35)
6	COL6A1	Collagen type VI alpha-1 chain		D		+	(36)
4	COL6A2	Collagen type VI alpha-2 chain		D		+	
29	COL6A3	Collagen type VI alpha-3 chain		D		+	
2	COPA	Coatomer subunit alpha	U	D	+		(37)
2	COPB1	Coatomer subunit beta		D	+		(38)
5	COPB2	Coatomer subunit beta’	U		+		(39)
2	COPZ1	Coatomer subunit zeta-1		D		+	
3	CORO1C	Coronin-1C				+	
4	CRK	Proto-oncogene C-crk	U	D		+	
5	CRTAP	Cartilage-associated protein, P3H5		D	+		
4	CSPG4	Chondroitin sulfate proteoglycan 4		D	+		(40)
3	CTSB	Cathepsin B, APP secretase	U	D		+	
2	CTSD	Cathepsin D	U	D		+	(41)
2	CUTA	CutA divalent cation tolerance homolog	U	D		+	
2	DBN1	Drebrin 1	U	D		+	(42)
3	DCN	Decorin		D	+		(43)
2	DCTN1	Dynactin subunit 1, 150 KDa Dynein-associated protein		D	+		(44)
5	DCTN2	Dynactin subunit 2				+	
12	DDB1	DNA damage-binding protein 1	U	D		+	(30)
2	DDX39	ATP-dependent RNA helicase DDX39A	U	D		+	
5	DDX39B	Spliceosome RNA helicase BAT1		D		+	
5	DHX15	ATP-dependent RNA helicase #46		D	+		
5	DHX9	ATP-dependent RNA helicase A			+		(45)
5	DIABLO	Diablo, IAP (Inihibitor of apoptosis protein)-binding	U			+	
2	DKC1	H/ACA ribonucleoprotein complex subunit DKC1	U	D	+		
2	DLST	Dihydrolipoyllysine-residue succinyltransferase component of 2- oxoglutarate dehydrogenase complex		D		+	(46)
2	DNAJB11	DnaJ (Hsp40) homolog subfamily B member 11	U			+	(47)
2	DPP3	Dipeptidyl-peptidase 3		D		+	
3	DPYSL2	Dihydropyrimidinase-related protein	U	D		+	(48)
3	DRG1	Developmentally-regulated GTP-binding protein		D		+	
5	DYNC1H1	Dynein cytoplasmic 1 heavy chain 1			+		
2	DYNC1I2	Dynein cytoplasmic 1 intermediate chain 2			+		
2	EEF1A1	Elongation factor 1-alph 1	U	D		+	(49)
3	EEF1A2	Elongation factor 1-alpha 2	U			+	(50)
2	EEF1B2	Elongation factor 1-beta 2		D		+	
5	EEF1D	Elongation factor 1-delta		D		+	
10	EEF1G	Elongation factor 1-gamma	U	D		+	
14	EEF2	Elongation factor 2	U	D		+	(51)
6	EFTUD2	116 kDa U5 snRNP component, SNRP116		D	+		(52)
4	EHD2	EH domain-containing protein 2	U	D		+	
3	EIF2S1	Eukaryotic translation initiation factor 2 subunit 1, EIF2A				+	(53)
10	EIF3A	Eukaryotic translation initiation factor 3 subunit A	U	D	+		(54)
9	EIF3B	Eukaryotic translation initiation factor 3 subunit B	U	D	+		
3	EIF3CL	Eukaryotic translation initiation factor 3 subunit C-like protein		D	+		
5	EIF3E	Eukaryotic translation initiation factor 3 subunit E	U	D	+		(55)
2	EIF3F	Eukaryotic translation initiation factor 3 subunit F	U	D	+		
2	EIF3G	Eukaryotic translation initiation factor 3 subunit G				+	
6	EIF3L	EIF3, subunit E interacting protein		D	+		
11	EIF4A1	Eukaryotic initiation factor 4A-1, DDX2A	U	D		+	
2	EIF4A3	Eukaryotic initiation factor 4A-III, DDX48				+	(56)
4	EIF4G1	Eukaryotic translation initiation factor 4 gamma 1	U	D		+	
2	EIF4G2	Eukaryotic translation initiation factor 4 gamma 2		D		+	
4	EIF5A	Eukaryotic translation initiation factor 5A-1	U	D		+	
2	EIF5A2	Eukaryotic translation initiation factor 5A-2		D		+	
3	EIF6	Eukaryotic translation initiation factor 6	U			+	
4	ELAVL1	ELAV-like protein		D		+	(57)
2	ELOB	Transcription elongation factor B, TCEB2	U	D		+	
2	ENO1	Alpha-enolase	U	D		+	(58)
7	ENO2	Gamma-enolase	U	D		+	(59)
2	ENOPH1	Enolase-phosphatase E1	U			+	
2	EPRS	Bifunctional aminoacyl-tRNA synthetase, EPRS1	U		+		(60)
6	ERP44	Endoplasmic reticulum resident protein ERp44				+	(61)
2	EWSR1	EWS RNA-binding protein	U			+	
2	FAF1	FAS-associated factor 1	U			+	
4	FAM62A	Extended synaptotagmin-1, ESYT1			+		(62)
2	FASN	Fatty acid synthase	U	D		+	(63)
3	FBLN1	Fibulin 1	U	D		+	(64)
8	FKBP10	FK506-binding protein 10				+	
4	FKBP9	FK506-binding protein 9		D		+	
43	FLNA	Filamin-A	U	D		+	(65)
8	FLNB	Filamin-B	U			+	(30)
24	FLNC	Filamin-C	U	D		+	(66)
23	FN1	Fibronectin	U	D		+	(67)
3	FSTL1	Follistatin-related protein	U	D		+	(68)
2	FTH1	Ferritin heavy chain	U	D		+	(68)
2	G6PD	Glucose-6-phosphate 1-dehydrogenase	U	D		+	
15	GANAB	Neutral alpha-glucosidase AB		D		+	(69)
2	GAPDH	Glyceraldehyde-3-phosphate dehydrogenase	U	D		+	(70)
2	GAR1	H/ACA ribonucleoprotein complex subunit 1			+		
2	GDI1	Rab GDP dissociation inhibitor alpha	U	D		+	(71)
2	GDI2	Rab GDP dissociation inhibitor beta	U	D		+	(72)
2	GLRX3	Glutaredoxin 3, Thioredoxin-like 2		D		+	(73)
2	GMFB	Glia maturation factor, beta	U			+	
5	GPC1	Glypican-1		D	+		
16	GSN	Gelsolin	U	D		+	(74)
4	GTF2I	General transcription factor II-I (GTF2IP4)	U	D		+	
2	H2AFV	Histone H2A.V, H2AZ2		D	+		(75)
4	H2AFY	Histone marcoH2A1, MAROH2A1	U		+		(76)
2	HARS	Histidyl-tRNA synthetase, cytoplasmic				+	(19)
3	HDGF	Hepatoma-derived growth factor	U	D		+	(77)
2	HDLBP	Vigilin, High density lipoprotein binding protein	U	D		+	
2	HEBP2	Heme-binding protein 2	U			+	
5	HEXB	Beta-hexosaminidase subunit beta		D		+	
4	HIST1H1B	Histone H1.5, H1–5	U	D		+	(78)
4	HIST1H1C	Histone H1.2, H1–2	U	D	+		(78)
2	HIST1H2BL	Histone H2B type 1-L, H2BC13	U	D	+		(79)
9	HIST1H4J	Histone H4, H4C1			+		(80)
11	HIST2H2BE	Histone H2B type 2-E, H2BC21	U	D	+		(81)
3	HIST2H3D	Histone H3.2, HIST2H3A, HIST2H3C, H3C13			+		(82)
4	HMGB1L1	High mobility group box 1 pseudogene 1, HMGB1 P1				+	(83)
2	HNRNPA1	U1 ribonucleoprotein A1	U	D		+	(84)
5	HNRNPA2B1	Putative uncharacterized protein HNRNPA2B1	U	D		+	(85)
2	HNRNPA3	Heterogeneous nuclear ribonucleoprotein A3	U	D		+	(86)
2	HNRNPC	Heterogeneous nuclear ribonucleoproteins C1/C2	U	D	+		(87)
7	HNRNPCL1	Heterogeneous nuclear ribonucleoprotein C-like 1				+	
2	HNRNPD	Heterogeneous nuclear ribonucleoprotein D, AUF1				+	(88)
3	HNRNPDL	Heterogeneous nuclear ribonucleoprotein D-like	U	D		+	(89)
5	HNRNPF	Heterogeneous nuclear ribonucleoprotein F		D		+	(90)
2	HNRNPH1	Heterogeneous nuclear ribonucleoprotein H1	U	D		+	(90)
2	HNRNPH3	Heterogeneous nuclear ribonucleoprotein H3	U	D		+	
9	HNRNPK	Heterogeneous nuclear ribonucleoprotein K	U			+	(91)
7	HNRNPR	Heterogeneous nuclear ribonucleoprotein R	U	D		+	(92)
5	HNRNPU	Heterogeneous nuclear ribonucleoprotein U	U	D		+	
3	HNRNPUL1	HnRNP U-like protein 1	U	D	+		
11	HSP90AA1	Heat shock 90kDa protein 1, alpha isoform	U	D		+	(93)
3	HSP90AA2	Putative heat shock protein HSP 90-alpha A				+	(94)
11	HSP90AB1	Heat shock protein HSP 90-beta	U	D		+	(95)
31	HSP90B1	Endoplasmin	U	D		+	(96)
3	HSPA1A	HSPA1B Heat shock 70 kDa protein 1A	U	D		+	
2	HSPA1L	Heat shock 70 kDa protein 1-like				+	(97)
2	HSPA4	Heat shock 70 kDa protein 4	U	D		+	
28	HSPA5	Endoplasmic reticulum chaperone BiP, GRP78	U	D		+	(98)
27	HSPA8	Heat shock cognate 71 kDa protein	U	D		+	(99)
8	HSPA9	Stress-70 protein, mitochondrial	U	D		+	(99)
7	HSPB1	Heat shock protein beta-1	U	D		+	(100)
2	HSPD1	60 kDa heat shock protein, mitochondrial	U	D		+	
3	HSPG2	Basement membrane heparan sulfate proteoglycan	U	D	+		(101)
2	HTATSF1	HIV Tat-specific factor 1		D	+		
7	HYOU1	Hypoxia up-regulated protein	U			+	
2	IGBP1	Immunoglobulin-binding protein 1	U	D		+	
7	ILF2	Interleukin enhancer-binding factor	U			+	(102)
2	ILF3	Interleukin enhancer-binding factor 3	U			+	(102)
13	IQGAP1	Ras GTPase-activating-like protein IQGAP1	U			+	(103)
2	IRGQ	Immunity-related GTPase family Q protein	U	D		+	
4	ITGB1	Integrin beta-1	U	D	+		
4	KARS	Lysyl-tRNA synthetase				+	(60)
2	KPNA3	Importin subunit alpha-4			+		
8	KPNB1	Importin subunit beta-1			+		(104)
10	KTN1	Kinectin	U			+	(105)
7	LAMB1	Laminin subunit beta-1		D		+	(106)
5	LAMC1	Laminin subunit gamma-1	U	D		+	(107)
3	LCP1	Plastin-2	U	D		+	(108)
5	LGALS1	Galectin-1	U	D		+	(109)
23	LMNA	Isoform A of Lamin-A/C	U	D		+	(110)
3	LMNB1	Lamin-B1	U	D		+	(111)
7	LMNB2	Lamin-B2	U	D	+		(112)
2	LRPPRC	Leucine-rich PPR motif-containing protein		D		+	(113)
2	LSM2	U6 snRNA-associated Sm-like protein LSm2	U			+	
2	LSM6	U6 snRNA-associated Sm-like protein LSm6	U			+	
2	MAGOHB	Protein mago nashi homolog	U	D		+	
3	MANBA	Beta-mannosidase		D		+	
3	MAP1B	Microtubule-associated protein 1 B	U	D		+	(114)
6	MAPRE1	Microtubule-associated protein RP/EB family member				+	
10	MOV10	Putative helicase, Moloney leukemia virus 10 protein	U	D	+		
3	MSN	Moesin	U			+	(115)
21	MVP	Major vault protein	U	D	+		(116)
4	MXRA5	Matrix-remodeling-associated protein 5		D	+		(116)
2	MYH10	Myosin-10	U	D	+		(117)
43	MYH9	Myosin-9	U	D	+		(117)
3	MYL6	Myosin light chain 6	U			+	
4	MYLK	Myosin light chain kinase, smooth muscle	U	D		+	
3	MYO1C	Unconventional myosin-Ic		D	+		(118)
2	NACA	Nascent polypeptide associated complex subunit alpha	U	D		+	(119)
3	NAP1L1	Nucleosome assembly protein 1-like 1	U	D	+		
3	NAP1L4	Nucleosome assembly protein 1-like 4	U	D	+		
2	NASP	Nuclear autoantigenic sperm protein	U	D		+	(120)
11	NCL	Nucleolin	U	D	+		(121)
2	NES	Nestin	U	D		+	
2	NEU1	Sialidase-1	U	D		+	(122)
3	NEXN	Nexilin F-actin binding protein	U	D		+	
2	NFU1	HIRA interacting protein 5				+	
3	NME1	Nucleoside diphosphate kinase A, RMRP	U	D		+	(123)
2	NMT1	Glycylpeptide N-tetradecanoyltransferase 1				+	(124)
2	NMT2	Glycylpeptide N-tetradecanoyltransferase 2		D		+	
4	NPEPPS	Puromycin-sensitive aminopeptidase				+	
7	NPM1	Nucleophosmin	U	D		+	(125)
5	NUDC	Nuclear distribution C, Dynein complex regulator		D		+	
3	NUDT21	Cleavage and polyadenylation specificity factor 5		D		+	
2	NUDT5	Nudix hydrolase 5		D		+	
3	NUMA1	Nuclear mitotic apparatus protein 1	U	D		+	(126)
5	P3H1	Basement membrane chondroitin sulfate proteoglycan	U			+	
2	P3H3	Prolyl 3-hydroxylase 3, LEPREL2		D	+		
2	P3H4	ER protein SC65, nucleolar autoantigen No55			+		(127)
2	P4HA2	Prolyl 4-hydroxylase subunit alpha-2		D		+	
18	P4HB	Protein disulfide-isomerase	U	D		+	(128)
4	PA2G4	Proliferation-associated protein 2G4	U	D		+	
19	PABPC1	Poly(A)-binding protein 1		D	+		(129)
7	PABPC4	Poly(A)-binding protein 4, APP1		D	+		(130)
3	PARVA	Alpha-parvin	U			+	
4	PCNA	Proliferating cell nuclear antigen	U	D		+	(131)
17	PDIA3	Protein disulfide-isomerase A3	U	D		+	(132)
34	PDIA4	Protein disulfide-isomerase A4	U	D		+	
9	PDIA6	Protein disulfide-isomerase A6	U	D		+	
3	PFDN2	Prefoldin subunit 2	U			+	(133)
8	PFN1	Profilin-1	U	D		+	(134)
2	PFN2	Profilin-2	U			+	(135)
91	PLEC	Plectin-1, PLEC1	U	D		+	(136)
5	PLOD1	Procollagen-lysine, 2-oxoglutarate 5-dioxygenase 1		D		+	
5	PLOD3	Multifunctional procollagen lysine hydroxylase and glycosyltransferase LH3				+	
6	PLS3	Plastin-3	U	D		+	
10	PPIB	Peptidyl-prolyl cis-trans isomerase	U	D		+	(137)
4	PRDX3	Thioredoxin-dependent peroxide reductase	U	D		+	(138)
3	PRDX4	Peroxiredoxin-4	U	D		+	(139)
2	PRKAR2A	Protein kinase CAMP-dependent type II regulatory alpha	U			+	
2	PRKCDBP	Protein kinase C delta-binding protein				+	
11	PRKCSH	Protein kinase C substrate 80K-H		D		+	
5	PRKDC	DNA-dependent protein kinase catalytic subunit	U	D	+		(140)
4	PRMT1	Protein arginine N-methyltransferase 1		D		+	
24	PRPF8	Pre-mRNA-processing-splicing factor 8	U	D	+		(30)
2	PSAP	Proactivator polypeptide, Prosaposin	U	D		+	
5	PSMA3	Proteasome subunit alpha type-3, C8	U	D		+	(141)
4	PSMA4	Proteasome subunit alpha type-4, C9	U			+	(142)
4	PSMA5	Proteasome subunit alpha type-5	U			+	(143)
6	PSMA6	Proteasome subunit alpha type-6	U	D		+	
6	PSMA7	Proteasome subunit alpha type-7	U	D		+	(144)
5	PSMB1	Proteasome subunit beta type-1				+	(145)
2	PSMB3	Proteasome subunit beta type-3		D		+	(141)
7	PSMB4	Proteasome subunit beta type-4				+	
3	PSMB6	Proteasome subunit beta type-6		D		+	
5	PSMB7	Proteasome subunit beta type-7		D		+	
2	PSMD1	26S proteasome non-ATPase regulatory subunit 1	U		+		
2	PSMD12	26S proteasome non-ATPase regulatory subunit 12		D	+		
3	PSMD13	Proteasome 26S non-ATPase subunit 13		D		+	(146)
9	PSMD6	26S proteasome non-ATPase regulatory subunit 6				+	
2	PSMD7	26S proteasome non-ATPase regulatory subunit 7	U			+	
6	PTBP1	Polypyrimidine tract-binding protein, hnRNP I	U	D		+	(147)
2	PTCD3	Pentatricopeptide repeat domain 3, MRPS39			+		
2	PUF60	Poly(U)-binding-splicing factor PUF60	U			+	(148)
2	PZP	Pregnancy zone protein, alpha-2-macroglobulin like		D		+	(149)
4	QARS	Glutaminyl-tRNA synthetase			+		(60)
3	RAB1A	Ras-related protein Rab-1A		D	+		
3	RAB7A	Ras-related protein Rab-7a	U	D		+	
3	RAD23A	UV excision repair protein RAD23 homolog A		D		+	(150)
5	RAD23B	UV excision repair protein RAD23 homolog B	U	D		+	(150)
6	RALY	RNA binding protein, autoantigen p542	U	D	+		(151)
5	RBBP4	Chromosome assembly factor 1 subunit C		D	+		(152)
2	RBM3	Putative RNA-binding protein 3	U	D		+	
2	RBMXL2	RNA-binding motif protein X-linked-like-2				+	
2	RCN3	Reticulocalbin-3				+	
2	RDX	Radixin				+	(153)
2	ROD1	Regulator of differentiation 1, PTBP3	U	D		+	(147)
2	RPF2	Ribosome production factor 2 homolog, BXDC1			+		
2	RPL11	60S ribosomal protein L11	U		+		
2	RPL12	60S ribosomal protein L12	U	D	+		(154)
2	RPL15	60S ribosomal protein L15		D	+		
3	RPL18	60S ribosomal protein L18		D	+		
2	RPL22	60S ribosomal protein L22		D		+	
16	RPL5	60S ribosomal protein L5		D	+		(155)
8	RPL6	60S ribosomal protein L6	U	D	+		(135)
8	RPL7	60S ribosomal protein L7	U	D	+		(156)
7	RPLP0	60S acidic ribosomal protein P0	U	D	+		(157)
4	RPLP2	60S acidic ribosomal protein P2	U	D	+		
3	RPS18	40S ribosomal protein S18	U	D	+		(158)
3	RPS19	40S ribosomal protein S19		D		+	(159)
3	RPS2	40S ribosomal protein S2	U	D	+		
4	RPS3	40S ribosomal protein S3	U	D		+	(160)
2	RPS3A	40S ribosomal protein S3a	U	D		+	
3	RPS4X	40S ribosomal protein S4, X isoform		D	+		
2	RPS8	40S ribosomal protein S8	U	D	+		
7	RPS9	40S ribosomal protein S9		D	+		(159)
13	RRBP1	Ribosome-binding protein 1	U	D		+	
2	SAE1	SUMO-activating enzyme subunit 1	U	D		+	(161)
4	SEPHS1	Selenide, water dikinase		D		+	(162)
2	SEPT2	Septin-2, NEDD5, DIFF6	U			+	(163)
3	SERPINE1	Plasminogen activator inhibitor 1	U	D		+	(164)
4	SERPINH1	Serpin H1, HSP47		D		+	(165)
6	SET	SET nuclear proto-oncogene	U	D	+		
6	SF3B1	Splicing factor 3B subunit 1	U	D	+		(166)
7	SF3B3	Splicing factor 3B subunit 3			+		(166)
3	SFPQ	Splicing factor, proline- and glutamine-rich	U	D		+	(167)
2	SFRS11	Splicing factor, arginine/serine-rich 11, SRSF11	U	D		+	
3	SFRS2	Splicing factor, arginine/serine-rich 2, SRSF2	U	D		+	(38)
2	SFRS7	Serine /arginine-rich splicing factor 7, SRSF7	U		+		(168)
3	SH3BGRL3	Putative uncharacterized protein, SH3 domain-binding glutamic acid-rich-like protein 3		D		+	
2	SKP1	S-phase kinase-associated protein 1	U	D		+	
2	SLC3A2	4F2 cell-surface antigen heavy chain, CD98	U	D	+		
4	SMS	Spermine synthase	U	D		+	
9	SND1	Staphylococcal nuclease domain-containing protein 1	U	D		+	
2	SNRNP200	U5 small nuclear ribonucleoprotein 200 kDa helicase		D	+		
3	SNRPA	U1 small nuclear ribonucleoprotein A	U			+	(169)
2	SNRPB	SnRNP-associated proteins B and B’	U	D	+		(170)
2	SNRPD1	Small nuclear ribonucleoprotein Sm D1	U		+		(171)
2	SNRPD2	Small nuclear ribonucleoprotein Sm D2		D	+		(172)
2	SNRPD3	Small nuclear ribonucleoprotein Sm D3		D	+		(171)
2	SNRPE	Small nuclear ribonucleoprotein E		D	+		(173)
37	SPTAN1	Highly similar to Spectrin alpha chain, brain	U	D		+	(174)
19	SPTBN1	Spectrin beta chain, brain	U	D	+		(175)
11	SSB	Lupus La protein	U			+	(19)
6	SSBP1	Single-stranded DNA-binding protein, mitochondrial			+		
4	SSRP1	FACT complex subunit SSRP1	U	D	+		(176)
3	ST13	Hsc70-interacting protein	U			+	(177)
2	STRBP	Spermatid perinuclear RNA-binding protein				+	
3	SUB1	Activated RNA polymerase II transcriptional coactivator p15	U	D		+	
2	SUMO1	Small ubiquitin-related modifier		D		+	(161)
4	SUPT16H	FACT complex subunit SPT16		D	+		
3	SYNCRIP	Heterogeneous nuclear ribonucleoprotein Q		D		+	
3	TFG	Trafficking from ER to Golgi regulator				+	
9	THBS1	Thrombospondin-1	U	D		+	(178)
29	TLN1	Talin-1	U	D		+	(179)
4	TLN2	Talin-2	U			+	
6	TNC	Tenascin C		D		+	(180)
3	TPD52L2	Tumor protein D54	U	D		+	
16	TPM1	Tropomyosin 1 alpha chain	U	D		+	(181)
17	TPM2	Tropomyosin beta chain	U	D		+	
6	TPM3	Tropomyosin alpha-3 chain	U	D		+	(182)
20	TPM4	Tropomyosin alpha-4 chain	U	D		+	(183)
2	TPP1	Tripeptidyl-peptidase 1	U	D	+		
4	TPR	Nucleoprotein TPR	U	D		+	(184)
4	TPT1	Tumor protein, translationally-controlled	U	D		+	
2	TROVE2	60 kDa SS-A/Ro ribonucleoprotein	U		+		
4	TUBA1C	Tubulin alpha-1C chain	U	D	+		(185)
6	TUBA4A	Tubulin alpha-4A chain, TUBA1	U	D	+		(186)
3	TUBB	Tubulin beta chain	U	D	+		(187)
2	TUBB1	Tubulin beta-1 chain			+		(186)
3	TUBB4B	Tubulin beta-2C, tubulin beta-4B, TUBB2C	U	D	+		(188)
2	TXN	Thioredoxin	U	D		+	(189)
2	TXNDC17	Thioredoxin domain-containing protein 17	U	D		+	
4	TXNDC5	Thioredoxin domain-containing protein 5	U	D		+	
2	TXNRD1	Thioredoxin reductase 1, cytoplasmic	U	D		+	(189)
8	UBA1	Ubiquitin-like modifier-activating enzyme 1	U			+	(190)
2	UCHL1	Ubiquitin carboxyl-terminal hydrolase isozyme L1	U	D		+	(191)
6	UGCGL1	UDP-glucose:glycoprotein glucosyltransferase 1		D		+	
18	UPF1	Regulator of nonsense transcripts 1		D	+		
3	USP5	Ubiquitin carboxyl-terminal hydrolase 5	U	D		+	
2	USP9X	Ubiquitin specific protease 9, X chromosome	U	D	+		
4	VASN	Vasorin	U	D		+	
4	VAT1	Synaptic vesicle membrane protein VAT-1 homolog	U	D		+	
3	VBP1	Von Hippel-Lindau binding protein		D		+	
13	VCL	Vinculin	U	D		+	(192)
15	VCP	Transitional endoplasmic reticulum ATPase	U	D		+	(193)
17	VIM	Vimentin	U	D	+		(194)
5	WARS	Tryptophanyl-tRNA synthetase, cytoplasmic	U	D		+	(195)
21	XRCC5	ATP-dependent DNA helicase 2 subunit 2, Ku80		D	+		(196)
21	XRCC6	ATP-dependent DNA helicase 2 subunit 1, Ku70	U	D	+		(197)
5	YBX3	D-binding protein A, CSDA, DBPA	U	D	+		(198)
5	YWHAB	14-3-3 protein beta/alpha	U	D		+	
9	YWHAE	14-3-3 protein epsilon	U	D		+	(199)
3	YWHAG	14-3-3 protein gamma	U	D		+	(199)
3	YWHAH	14-3-3 protein eta		D		+	(200)
5	YWHAQ	14-3-3 protein theta	U	D		+	(201)
5	YWHAZ	14-3-3 protein zeta/delta	U	D		+	(202)

References for Table 1

1. R. D. Saunders, S. T. Nakajima, S. N. Rai, J. Pan, C. Gercel-Taylor, D. D. Taylor, Alterations in antibody subclass immune reactivity to trophoblast-derived fetal fibronectin and α2-macroglobulin in women with recurrent pregnancy loss. *Am J Reprod Immunol* **68**, 438–449 (2012).

2. C. C. Bunn, R. M. Bernstein, M. B. Mathews, Autoantibodies against alanyl-tRNA synthetase and tRNAAla coexist and are associated with myositis. *The Journal of experimental medicine* **163**, 1281–1291 (1986).

3. P. V. Mande, F. R. Parikh, I. Hinduja, K. Zaveri, R. Vaidya, R. Gajbhiye, V. V. Khole, Identification and validation of candidate biomarkers involved in human ovarian autoimmunity. *Reprod Biomed Online* **23**, 471–483 (2011).

4. J. J. van Beers, C. M. Schwarte, J. Stammen-Vogelzangs, E. Oosterink, B. Bozic, G. J. Pruijn, The rheumatoid arthritis synovial fluid citrullinome reveals novel citrullinated epitopes in apolipoprotein E, myeloid nuclear differentiation antigen, and beta-actin. *Arthritis and rheumatism* **65**, 69–80 (2013).

5. C. Hanrotel-Saliou, I. Segalen, Y. Le Meur, P. Youinou, Y. Renaudineau, Glomerular antibodies in lupus nephritis. *Clin Rev Allergy Immunol* **40**, 151–158 (2011).

6. T. Wang, M. Liu, S. J. Zheng, D. D. Bian, J. Y. Zhang, J. Yao, Q. F. Zheng, A. M. Shi, W. H. Li, L. Li, Y. Chen, J. H. Wang, Z. P. Duan, L. Dong, Tumor-associated autoantibodies are useful biomarkers in immunodiagnosis of α-fetoprotein-negative hepatocellular carcinoma. *World J Gastroenterol* **23**, 3496–3504 (2017).

7. F. Sköldberg, L. Rönnblom, M. Thornemo, A. Lindahl, P. I. Bird, F. Rorsman, O. Kämpe, E. Landgren, Identification of AHNAK as a novel autoantigen in systemic lupus erythematosus. *Biochemical and biophysical research communications* **291**, 951–958 (2002).

8. J. Nehring, L. A. Schirmbeck, J. Friebus-Kardash, D. Dubler, U. Huynh-Do, C. Chizzolini, C. Ribi, M. Trendelenburg, Autoantibodies Against Albumin in Patients With Systemic Lupus Erythematosus. *Frontiers in immunology* **9**, 2090 (2018).

9. D. Lu, E. Kuhn, R. E. Bristow, R. L. Giuntoli, 2nd, S. K. Kjær, M. Shih Ie, R. B. Roden, Comparison of candidate serologic markers for type I and type II ovarian cancer. *Gynecol Oncol* **122**, 560–566 (2011).

10. D. J. Caster, E. A. Korte, M. L. Merchant, J. B. Klein, D. W. Wilkey, B. H. Rovin, D. J. Birmingham, J. B. Harley, B. L. Cobb, B. Namjou, K. R. McLeish, D. W. Powell, Autoantibodies targeting glomerular annexin A2 identify patients with proliferative lupus nephritis. *Proteomics Clin Appl* **9**, 1012–1020 (2015).

11. A. Hrycek, P. Cieślik, Annexin A5 and anti-annexin antibodies in patients with systemic lupus erythematosus. *Rheumatol Int* **32**, 1335–1342 (2012).

12. Y. Seko, A. Matsumoto, T. Fukuda, Y. Imai, T. Fujimura, H. Taka, R. Mineki, K. Murayama, Y. Hirata, R. Nagai, A case of neonatal lupus erythematosus presenting delayed dilated cardiomyopathy with circulating autoantibody to annexin A6. *Int Heart J* **48**, 407–415 (2007).

13. S. Jarius, B. Wildemann, ‘Medusa head ataxia’: the expanding spectrum of Purkinje cell antibodies in autoimmune cerebellar ataxia. Part 3: Anti-Yo/CDR2, anti-Nb/AP3B2, PCA-2, anti-Tr/DNER, other antibodies, diagnostic pitfalls, summary and outlook. *Journal of neuroinflammation* **12**, 168 (2015).

14. N. Vuilleumier, F. Montecucco, O. Hartley, Autoantibodies to apolipoprotein A-1 as a biomarker of cardiovascular autoimmunity. *World J Cardiol* **6**, 314–326 (2014).

15. J. Creaney, I. M. Dick, D. Yeoman, S. Wong, B. W. Robinson, Auto-antibodies to β-F1-ATPase and vimentin in malignant mesothelioma. *PloS one* **6**, e26515 (2011).

16. A. Polgár, A. Falus, E. Koó, I. Ujfalussy, M. Seszták, I. Szuts, K. Konrád, L. Hodinka, E. Bene, G. Mészáros, Z. Ortutay, E. Farkas, A. Paksy, E. I. Buzás, Elevated levels of synovial fluid antibodies reactive with the small proteoglycans biglycan and decorin in patients with rheumatoid arthritis or other joint diseases. *Rheumatology (Oxford, England)* **42**, 522–527 (2003).

17. N. M. Bhat, C. M. Adams, Y. Chen, M. M. Bieber, N. N. Teng, Identification of Cell Surface Straight Chain Poly-N-Acetyl-Lactosamine Bearing Protein Ligands for VH4–34-Encoded Natural IgM Antibodies. *Journal of immunology (Baltimore, Md. : 1950)* **195**, 5178–5188 (2015).

18. V. M. Beutgen, C. Schmelter, N. Pfeiffer, F. H. Grus, Autoantigens in the trabecular meshwork and glaucoma-specific alterations in the natural autoantibody repertoire. *Clin Transl Immunology* **9**, e01101 (2020).

19. C. N. Gruber, R. S. Patel, R. Trachtman, L. Lepow, F. Amanat, F. Krammer, K. M. Wilson, K. Onel, D. Geanon, K. Tuballes, M. Patel, K. Mouskas, T. O’Donnell, E. Merritt, N. W. Simons, V. Barcessat, D. M. Del Valle, S. Udondem, G. Kang, S. Gangadharan, G. Ofori-Amanfo, U. Laserson, A. Rahman, S. Kim-Schulze, A. W. Charney, S. Gnjatic, B. D. Gelb, M. Merad, D. Bogunovic, Mapping Systemic Inflammation and Antibody Responses in Multisystem Inflammatory Syndrome in Children (MIS-C). *Cell* **183**, 982–995.e914 (2020).

20. U. Kishore, R. D. Sontheimer, K. N. Sastry, E. G. Zappi, G. R. Hughes, M. A. Khamashta, K. B. Reid, P. Eggleton, The systemic lupus erythematosus (SLE) disease autoantigen-calreticulin can inhibit C1q association with immune complexes. *Clinical and experimental immunology* **108**, 181–190 (1997).

21. B. Terrier, M. C. Tamby, L. Camoin, P. Guilpain, C. Broussard, G. Bussone, A. Yaïci, F. Hotellier, G. Simonneau, L. Guillevin, M. Humbert, L. Mouthon, Identification of target antigens of antifibroblast antibodies in pulmonary arterial hypertension. *American journal of respiratory and critical care medicine* **177**, 1128–1134 (2008).

22. C. K. Weber, M. Haslbeck, M. Englbrecht, B. Sehnert, D. Mielenz, D. Graef, J. H. Distler, R. B. Mueller, H. Burkhardt, G. Schett, R. E. Voll, B. G. Furnrohr, Antibodies to the endoplasmic reticulum-resident chaperones calnexin, BiP and Grp94 in patients with rheumatoid arthritis and systemic lupus erythematosus. *Rheumatology (Oxford, England)* **49**, 2255–2263 (2010).

23. K. Matsuo, Y. Xiang, H. Nakamura, K. Masuko, K. Yudoh, K. Noyori, K. Nishioka, T. Saito, T. Kato, Identification of novel citrullinated autoantigens of synovium in rheumatoid arthritis using a proteomic approach. *Arthritis research & therapy* **8**, R175 (2006).

24. W. H. Li, J. Zhao, H. Y. Li, H. Liu, A. L. Li, H. X. Wang, J. Wang, K. He, B. Liang, M. Yu, B. F. Shen, X. M. Zhang, Proteomics-based identification of autoantibodies in the sera of healthy Chinese individuals from Beijing. *Proteomics* **6**, 4781–4789 (2006).

25. K. Furuta, B. Hildebrandt, S. Matsuoka, K. Kiyosawa, G. Reimer, C. Luderschmidt, E. K. Chan, E. M. Tan, Immunological characterization of heterochromatin protein p25beta autoantibodies and relationship with centromere autoantibodies and pulmonary fibrosis in systemic scleroderma. *J Mol Med (Berl)* **76**, 54–60 (1998).

26. K. Ohyama, M. Baba, M. Tamai, N. Aibara, K. Ichinose, N. Kishikawa, A. Kawakami, N. Kuroda, Proteomic profiling of antigens in circulating immune complexes associated with each of seven autoimmune diseases. *Clin Biochem* **48**, 181–185 (2015).

27. K. Hirai, H. Maeda, K. Omori, T. Yamamoto, S. Kokeguchi, S. Takashiba, Serum antibody response to group II chaperonin from Methanobrevibacter oralis and human chaperonin CCT. *Pathog Dis* **68**, 12–19 (2013).

28. M. Ebrahimi, E. Nylander, B. Bäcklund, Y. B. Wahlin, P. J. Coates, K. Nylander, The use of a novel ELISA method for detection of antibodies against p63 in sera from patients diagnosed with oral and/or genital and skin lichen planus. *Journal of oral pathology & medicine : official publication of the International Association of Oral Pathologists and the American Academy of Oral Pathology* **39**, 486–490 (2010).

29. L. Zhu, W. Shen, M. Zhu, N. J. Coorey, A. P. Nguyen, D. Barthelmes, M. C. Gillies, Anti-retinal antibodies in patients with macular telangiectasia type 2. *Invest Ophthalmol Vis Sci* **54**, 5675–5683 (2013).

30. J. H. Rho, W. Zhang, M. Murali, M. H. Roehrl, J. Y. Wang, Human proteins with affinity for dermatan sulfate have the propensity to become autoantigens. *Am J Pathol* **178**, 2177–2190 (2011).

31. M. K. Koivula, S. Aman, A. Karjalainen, M. Hakala, J. Risteli, Are there autoantibodies reacting against citrullinated peptides derived from type I and type II collagens in patients with rheumatoid arthritis? *Annals of the rheumatic diseases* **64**, 1443–1450 (2005).

32. J. Pardos-Gea, J. Cortés-Hernández, J. Castro-Marrero, E. Balada, J. Ordi-Ros, Autoantibodies to types I and IV collagen and heart valve disease in systemic lupus erythematosus/antiphospholipid syndrome. *Clinical rheumatology* **36**, 1401–1406 (2017).

33. G. R. Araujo, J. E. Fonseca, P. T. Fujimura, J. P. Cunha-Junior, C. H. Silva, A. F. Mourão, H. Canhão, L. R. Goulart, J. Gonçalves, C. Ueira-Vieira, Anti-type II collagen antibodies detection and avidity in patients with oligoarticular and polyarticular forms of juvenile idiopathic arthritis. *Immunology letters* **165**, 20–25 (2015).

34. G. Nakos, A. Adams, N. Andriopoulos, Antibodies to collagen in patients with idiopathic pulmonary fibrosis. *Chest* **103**, 1051–1058 (1993).

35. R. R. Hachem, V. Tiriveedhi, G. A. Patterson, A. Aloush, E. P. Trulock, T. Mohanakumar, Antibodies to K-α 1 tubulin and collagen V are associated with chronic rejection after lung transplantation. *American journal of transplantation : official journal of the American Society of Transplantation and the American Society of Transplant Surgeons* **12**, 2164–2171 (2012).

36. D. S. Nath, H. I. Basha, T. Mohanakumar, Antihuman leukocyte antigen antibody-induced autoimmunity: role in chronic rejection. *Current opinion in organ transplantation* **15**, 16–20 (2010).

37. T. J. Vece, L. B. Watkin, S. Nicholas, D. Canter, M. C. Braun, R. P. Guillerman, K. W. Eldin, G. Bertolet, S. McKinley, M. de Guzman, L. Forbes, I. Chinn, J. S. Orange, Copa Syndrome: a Novel Autosomal Dominant Immune Dysregulatory Disease. *J Clin Immunol* **36**, 377–387 (2016).

38. Q. Yang, J. Qin, G. Sun, C. Qiu, D. Jiang, H. Ye, X. Wang, L. Dai, J. Zhu, P. Wang, J. Zhang, Discovery and Validation of Serum Autoantibodies Against Tumor-Associated Antigens as Biomarkers in Gastric Adenocarcinoma Based on the Focused Protein Arrays. *Clin Transl Gastroenterol* **12**, e00284 (2020).

39. H. S. Hong, W. H. Chung, S. I. Hung, M. J. Chen, S. H. Lee, L. C. Yang, Clinical association of anti-golgi autoantibodies and their autoantigens. *Scand J Immunol* **59**, 79–87 (2004).

40. R. Dummer, A. Mittelman, F. P. Fanizzi, G. Lucchese, J. Willers, D. Kanduc, Non-self-discrimination as a driving concept in the identification of an immunodominant HMW-MAA epitopic peptide sequence by autoantibodies from melanoma cancer patients. *Int J Cancer* **111**, 720–726 (2004).

41. V. Vetvicka, M. Fusek, Cathepsin D: Autoantibody profiling as a diagnostic marker for cancers. *World J Clin Oncol* **4**, 1–3 (2013).

42. J. Pitsch, D. Kamalizade, A. Braun, J. C. Kuehn, P. E. Gulakova, T. Rüber, G. Lubec, D. Dietrich, R. von Wrede, C. Helmstaedter, R. Surges, C. E. Elger, E. Hattingen, H. Vatter, S. Schoch, A. J. Becker, Drebrin Autoantibodies in Patients with Seizures and Suspected Encephalitis. *Ann Neurol* **87**, 869–884 (2020).

43. C. A. Brandsma, H. A. Kerstjens, W. H. van Geffen, M. Geerlings, D. S. Postma, M. N. Hylkema, W. Timens, Differential switching to IgG and IgA in active smoking COPD patients and healthy controls. *Eur Respir J* **40**, 313–321 (2012).

44. M. J. Fritzler, J. C. Hamel, R. L. Ochs, E. K. Chan, Molecular characterization of two human autoantigens: unique cDNAs encoding 95- and 160-kD proteins of a putative family in the Golgi complex. *The Journal of experimental medicine* **178**, 49–62 (1993).

45. R. H. Scofield, Do we need new autoantibodies in lupus? *Arthritis research & therapy* **12**, 120 (2010).

46. D. R. Fregeau, T. Prindiville, R. L. Coppel, M. Kaplan, E. R. Dickson, M. E. Gershwin, Inhibition of alpha-ketoglutarate dehydrogenase activity by a distinct population of autoantibodies recognizing dihydrolipoamide succinyltransferase in primary biliary cirrhosis. *Hepatology* **11**, 975–981 (1990).

47. M. Oka, S. Sato, H. Soda, M. Fukuda, S. Kawabata, K. Nakatomi, K. Shiozawa, Y. Nakamura, K. Ohtsuka, S. Kohno, Autoantibody to heat shock protein Hsp40 in sera of lung cancer patients. *Japanese journal of cancer research : Gann* **92**, 316–320 (2001).

48. M. M. Harper, D. Rudd, K. J. Meyer, A. G. Kanthasamy, V. Anantharam, A. A. Pieper, E. Vázquez-Rosa, M. K. Shin, K. Chaubey, Y. Koh, L. P. Evans, A. G. Bassuk, M. G. Anderson, L. Dutca, I. T. Kudva, M. John, Identification of chronic brain protein changes and protein targets of serum auto-antibodies after blast-mediated traumatic brain injury. *Heliyon* **6**, e03374 (2020).

49. E. G. Kim, S. H. Kwak, D. Hwang, E. C. Yi, K. S. Park, B. K. Koo, K. M. Kim, The Level of Autoantibodies Targeting Eukaryote Translation Elongation Factor 1 α1 and Ubiquitin-Conjugating Enzyme 2L3 in Nondiabetic Young Adults. *Diabetes Metab J* **40**, 154–160 (2016).

50. C. J. Mooney, E. J. Dunphy, B. Stone, D. G. McNeel, Identification of autoantibodies elicited in a patient with prostate cancer presenting as dermatomyositis. *Int J Urol* **13**, 211–217 (2006).

51. F. Fernández-Madrid, N. Tang, H. Alansari, J. L. Granda, L. Tait, K. C. Amirikia, M. Moroianu, X. Wang, R. L. Karvonen, Autoantibodies to Annexin XI-A and Other Autoantigens in the Diagnosis of Breast Cancer. *Cancer research* **64**, 5089–5096 (2004).

52. M. Bach, G. Winkelmann, R. Luhrmann, 20S small nuclear ribonucleoprotein U5 shows a surprisingly complex protein composition. *Proceedings of the National Academy of Sciences of the United States of America* **86**, 6038–6042 (1989).

53. E. A. Waterman, D. J. Gawkrodger, P. F. Watson, A. P. Weetman, E. H. Kemp, Autoantigens in vitiligo identified by the serological selection of a phage-displayed melanocyte cDNA expression library. *The Journal of investigative dermatology* **130**, 230–240 (2010).

54. C. K. Heo, H. M. Hwang, H. J. Lee, S. S. Kwak, J. S. Yoo, D. Y. Yu, K. J. Lim, S. Lee, E. W. Cho, Serum anti-EIF3A autoantibody as a potential diagnostic marker for hepatocellular carcinoma. *Sci Rep* **9**, 11059 (2019).

55. Z. Betteridge, H. Chinoy, J. Vencovsky, J. Winer, K. Putchakayala, P. Ho, I. Lundberg, K. Danko, R. Cooper, N. McHugh, Identification of a novel autoantigen eukaryotic initiation factor 3 associated with polymyositis. *Rheumatology (Oxford, England)* **59**, 1026–1030 (2020).

56. G. Suwarnalata, A. H. Tan, H. Isa, R. Gudimella, A. Anwar, M. F. Loke, S. Mahadeva, S. Y. Lim, J. Vadivelu, Augmentation of Autoantibodies by Helicobacter pylori in Parkinson’s Disease Patients May Be Linked to Greater Severity. *PloS one* **11**, e0153725 (2016).

57. L. B. Nabors, H. M. Furneaux, P. H. King, HuR, a novel target of anti-Hu antibodies, is expressed in non-neural tissues. *J Neuroimmunol* **92**, 152–159 (1998).

58. S. Moscato, F. Pratesi, A. Sabbatini, D. Chimenti, M. Scavuzzo, R. Passatino, S. Bombardieri, A. Giallongo, P. Migliorini, Surface expression of a glycolytic enzyme, alpha-enolase, recognized by autoantibodies in connective tissue disorders. *Eur J Immunol* **30**, 3575–3584 (2000).

59. D. T. O’Dwyer, V. Clifton, A. Hall, R. Smith, P. J. Robinson, P. A. Crock, Pituitary autoantibodies in lymphocytic hypophysitis target both gamma- and alpha-Enolase - a link with pregnancy? *Archives of physiology and biochemistry* **110**, 94–98 (2002).

60. I. N. Targoff, E. P. Trieu, F. W. Miller, Reaction of anti-OJ autoantibodies with components of the multi-enzyme complex of aminoacyl-tRNA synthetases in addition to isoleucyl-tRNA synthetase. *The Journal of clinical investigation* **91**, 2556–2564 (1993).

61. M. Garranzo-Asensio, P. San Segundo-Acosta, C. Povés, M. J. Fernández-Aceñero, J. Martínez-Useros, A. Montero-Calle, G. Solís-Fernández, M. Sanchez-Martinez, N. Rodríguez, M. Cerón, S. Fernandez-Diez, G. Domínguez, V. de Los Ríos, A. Peláez-García, A. Guzmán-Aránguez, R. Barderas, Identification of tumor-associated antigens with diagnostic ability of colorectal cancer by in-depth immunomic and seroproteomic analysis. *Journal of proteomics* **214**, 103635 (2020).

62. C. Leveque, T. Hoshino, P. David, Y. Shoji-Kasai, K. Leys, A. Omori, B. Lang, O. el Far, K. Sato, N. Martin-Moutot, et al., The synaptic vesicle protein synaptotagmin associates with calcium channels and is a putative Lambert-Eaton myasthenic syndrome antigen. *Proceedings of the National Academy of Sciences of the United States of America* **89**, 3625–3629 (1992).

63. C. K. Heo, M. K. Woo, D. Y. Yu, J. Y. Lee, J. S. Yoo, H. S. Yoo, J. H. Ko, J. M. Kim, J. Y. Choi, I. G. Kim, S. G. Paik, E. W. Cho, Identification of autoantibody against fatty acid synthase in hepatocellular carcinoma mouse model and its application to diagnosis of HCC. *Int J Oncol* **36**, 1453–1459 (2010).

64. S. Forti, M. J. Scanlan, A. Invernizzi, F. Castiglioni, S. Pupa, R. Agresti, R. Fontanelli, D. Morelli, L. J. Old, S. M. Pupa, S. Ménard, Identification of breast cancer-restricted antigens by antibody screening of SKBR3 cDNA library using a preselected patient’s serum. *Breast cancer research and treatment* **73**, 245–256 (2002).

65. J. Kamhieh-Milz, V. Sterzer, H. Celik, O. Khorramshahi, R. Fadl Hassan Moftah, A. Salama, Identification of novel autoantigens via mass spectroscopy-based antibody-mediated identification of autoantigens (MS-AMIDA) using immune thrombocytopenic purpura (ITP) as a model disease. *Journal of proteomics* **157**, 59–70 (2017).

66. M. Adachi-Hayama, A. Adachi, N. Shinozaki, T. Matsutani, T. Hiwasa, M. Takiguchi, N. Saeki, Y. Iwadate, Circulating anti-filamin C autoantibody as a potential serum biomarker for low-grade gliomas. *BMC Cancer* **14**, 452 (2014).

67. W. Y. Wang, C. W. Twu, Y. C. Liu, H. H. Lin, C. J. Chen, J. C. Lin, Fibronectin promotes nasopharyngeal cancer cell motility and proliferation. *Biomed Pharmacother* **109**, 1772–1784 (2019).

68. X. Dong, M. Yang, H. Sun, J. Lü, Z. Zheng, Z. Li, L. Zhong, Combined measurement of CA 15–3 with novel autoantibodies improves diagnostic accuracy for breast cancer. *Onco Targets Ther* **6**, 273–279 (2013).

69. Y. Kit, M. Starykovych, M. Vajrychova, J. Lenco, D. Zastavna, R. Stoika, Detection of novel autoantigens in patients with recurrent miscarriage: description of an approach and preliminary findings. *Croat Med J* **55**, 259–264 (2014).

70. F. Delunardo, D. Soldati, V. Bellisario, A. Berry, S. Camerini, M. Crescenzi, C. Alessandri, F. Conti, F. Ceccarelli, A. Francia, G. Valesini, F. Cirulli, A. Siracusano, A. Siracusano, C. Niolu, I. Alex Rubino, E. Ortona, P. Margutti, Anti-GAPDH Autoantibodies as a Pathogenic Determinant and Potential Biomarker of Neuropsychiatric Diseases. *Arthritis Rheumatol* **68**, 2708–2716 (2016).

71. A. Kiyota, S. Iwama, Y. Sugimura, S. Takeuchi, H. Takagi, N. Iwata, K. Nakashima, H. Suzuki, T. Nishioka, T. Kato, A. Enomoto, H. Arima, K. Kaibuchi, Y. Oiso, Identification of the novel autoantigen candidate Rab GDP dissociation inhibitor alpha in isolated adrenocorticotropin deficiency. *Endocrine journal* **62**, 153–160 (2015).

72. O. Massa, M. Alessio, L. Russo, G. Nardo, V. Bonetto, F. Bertuzzi, A. Paladini, D. Iafusco, P. Patera, G. Federici, T. Not, C. Tiberti, R. Bonfanti, F. Barbetti, Serological Proteome Analysis (SERPA) as a tool for the identification of new candidate autoantigens in type 1 diabetes. *Journal of proteomics* **82**, 263–273 (2013).

73. J. M. Chung, Y. Jung, Y. P. Kim, J. Song, S. Kim, J. Y. Kim, M. Kwon, J. H. Yoon, M. D. Kim, J. K. Lee, D. Y. Chung, S. Y. Lee, J. Kang, H. C. Kang, Identification of the Thioredoxin-Like 2 Autoantibody as a Specific Biomarker for Triple-Negative Breast Cancer. *Journal of breast cancer* **21**, 87–90 (2018).

74. S. Biswas, S. Sharma, A. Saroha, D. S. Bhakuni, R. Malhotra, M. Zahur, M. Oellerich, H. R. Das, A. R. Asif, Identification of novel autoantigen in the synovial fluid of rheumatoid arthritis patients using an immunoproteomics approach. *PloS one* **8**, e56246 (2013).

75. R. L. Rubin, S. A. Bell, R. W. Burlingame, Autoantibodies associated with lupus induced by diverse drugs target a similar epitope in the (H2A-H2B)-DNA complex. *The Journal of clinical investigation* **90**, 165–173 (1992).

76. R. W. Burlingame, M. L. Boey, G. Starkebaum, R. L. Rubin, The central role of chromatin in autoimmune responses to histones and DNA in systemic lupus erythematosus. *The Journal of clinical investigation* **94**, 184–192 (1994).

77. H. Nahamura, K. Yoshida, Y. Kishima, H. Enomoto, H. Uyama, T. Kuroda, Y. Okuda, T. Hirotani, H. Ito, I. Kawase, Circulating auto-antibody against hepatoma-derived growth factor (HDGF) in patients with ulcerative colitis. *Hepatogastroenterology* **51**, 470–475 (2004).

78. J. Wesierska-Gadek, E. Penner, H. Lindner, E. Hitchman, G. Sauermann, Autoantibodies against different histone H1 subtypes in systemic lupus erythematosus sera. *Arthritis and rheumatism* **33**, 1273–1278 (1990).

79. S. V. Baranova, P. S. Dmitrienok, N. V. Ivanisenko, V. N. Buneva, G. A. Nevinsky, Antibodies to H2a and H2b histones from the sera of HIV-infected patients catalyze site-specific degradation of these histones. *Molecular bioSystems* **13**, 1090–1101 (2017).

80. M. Bruschi, M. Galetti, R. A. Sinico, G. Moroni, A. Bonanni, A. Radice, A. Tincani, F. Pratesi, P. Migliorini, C. Murtas, F. Franceschini, B. Trezzi, F. Brunini, R. Gatti, R. Tardanico, G. Barbano, G. Piaggio, P. Messa, P. Ravani, F. Scolari, G. Candiano, A. Martini, L. Allegri, G. M. Ghiggeri, Glomerular Autoimmune Multicomponents of Human Lupus Nephritis In Vivo (2): Planted Antigens. *J Am Soc Nephrol* **26**, 1905–1924 (2015).

81. C. C. van Bavel, J. Dieker, S. Muller, J. P. Briand, M. Monestier, J. H. Berden, J. van der Vlag, Apoptosis-associated acetylation on histone H2B is an epitope for lupus autoantibodies. *Molecular immunology* **47**, 511–516 (2009).

82. S. V. Baranova, P. S. Dmitrenok, A. D. Zubkova, N. V. Ivanisenko, E. S. Odintsova, V. N. Buneva, G. A. Nevinsky, Antibodies against H3 and H4 histones from the sera of HIV-infected patients catalyze site-specific degradation of these histones. *Journal of molecular recognition : JMR* **31**, e2703 (2018).

83. S. Barnay-Verdier, L. Fattoum, C. Borde, S. Kaveri, S. Gibot, V. Maréchal, Emergence of autoantibodies to HMGB1 is associated with survival in patients with septic shock. *Intensive care medicine* **37**, 957–962 (2011).

84. C. Guarneri, M. Aguennouz, F. Guarneri, F. Polito, S. Benvenga, S. P. Cannavo, Autoimmunity to heterogeneous nuclear ribonucleoprotein A1 in psoriatic patients and correlation with disease severity. *J Dtsch Dermatol Ges* **16**, 1103–1107 (2018).

85. M. F. Konig, J. T. Giles, P. A. Nigrovic, F. Andrade, Antibodies to native and citrullinated RA33 (hnRNP A2/B1) challenge citrullination as the inciting principle underlying loss of tolerance in rheumatoid arthritis. *Annals of the rheumatic diseases* **75**, 2022–2028 (2016).

86. S. Siapka, M. Patrinou-Georgoula, P. G. Vlachoyiannopoulos, A. Guialis, Multiple specificities of autoantibodies against hnRNP A/B proteins in systemic rheumatic diseases and hnRNP L as an associated novel autoantigen. *Autoimmunity* **40**, 223–233 (2007).

87. N. H. Heegaard, M. R. Larsen, T. Muncrief, A. Wiik, P. Roepstorff, Heterogeneous nuclear ribonucleoproteins C1/C2 identified as autoantigens by biochemical and mass spectrometric methods. *Arthritis research* **2**, 407–414 (2000).

88. K. Skriner, W. Hueber, E. Süleymanoglu, E. Höfler, V. Krenn, J. Smolen, G. Steiner, AUF1, the regulator of tumor necrosis factor alpha messenger RNA decay, is targeted by autoantibodies of patients with systemic rheumatic diseases. *Arthritis and rheumatism* **58**, 511–520 (2008).

89. Y. Zhang, H. Zhao, B. Liu, L. Li, L. Zhang, M. Bao, X. Ji, X. He, J. Yi, P. Chen, C. Lu, A. Lu, Low Level Antibodies Against Alpha-Tropomyosin Are Associated With Increased Risk of Coronary Heart Disease. *Frontiers in pharmacology* **11**, 195 (2020).

90. K. Op De Beéck, L. Maes, K. Van den Bergh, R. Derua, E. Waelkens, K. Van Steen, P. Vermeersch, R. Westhovens, K. De Vlam, P. Verschueren, H. Hooijkaas, D. Blockmans, X. Bossuyt, Heterogeneous nuclear RNPs as targets of autoantibodies in systemic rheumatic diseases. *Arthritis and rheumatism* **64**, 213–221 (2012).

91. L. Yang, M. Fujimoto, H. Murota, S. Serada, M. Fujimoto, H. Honda, K. Yamada, K. Suzuki, A. Nishikawa, Y. Hosono, Y. Yoneda, K. Takehara, Y. Imura, T. Mimori, T. Takeuchi, I. Katayama, T. Naka, Proteomic identification of heterogeneous nuclear ribonucleoprotein K as a novel cold-associated autoantigen in patients with secondary Raynaud’s phenomenon. *Rheumatology (Oxford, England)* **54**, 349–358 (2015).

92. W. Hassfeld, E. K. Chan, D. A. Mathison, D. Portman, G. Dreyfuss, G. Steiner, E. M. Tan, Molecular definition of heterogeneous nuclear ribonucleoprotein R (hnRNP R) using autoimmune antibody: immunological relationship with hnRNP P. *Nucleic Acids Res* **26**, 439–445 (1998).

93. L. Harlow, I. O. Rosas, B. R. Gochuico, T. R. Mikuls, P. F. Dellaripa, C. V. Oddis, D. P. Ascherman, Identification of citrullinated hsp90 isoforms as novel autoantigens in rheumatoid arthritisassociated interstitial lung disease. *Arthritis and rheumatism* **65**, 869–879 (2013).

94. H. Y. Qin, J. L. Mahon, M. A. Atkinson, P. Chaturvedi, E. Lee-Chan, B. Singh, Type 1 diabetes alters anti-hsp90 autoantibody isotype. *J Autoimmun* **20**, 237–245 (2003).

95. C. Cid, I. Regidor, A. Alcazar, Anti-heat shock protein 90beta antibodies are detected in patients with multiple sclerosis during remission. *J Neuroimmunol* **184**, 223–226 (2007).

96. S. Suzuki, K. Utsugisawa, K. Iwasa, T. Satoh, Y. Nagane, H. Yoshikawa, M. Kuwana, N. Suzuki, Autoimmunity to endoplasmic reticulum chaperone GRP94 in myasthenia gravis. *J Neuroimmunol* **237**, 87–92 (2011).

97. M. Chen, F. Aosai, H. S. Mun, K. Norose, H. Hata, A. Yano, Anti-HSP70 autoantibody formation by B1 cells in Toxoplasma gondii-infected mice. *Infect Immun* **68**, 4893–4899 (2000).

98. F. Shimizu, K. L. Schaller, G. P. Owens, A. C. Cotleur, D. Kellner, Y. Takeshita, B. Obermeier, T. J. Kryzer, Y. Sano, T. Kanda, V. A. Lennon, R. M. Ransohoff, J. L. Bennett, Glucose-regulated protein 78 autoantibody associates with blood-brain barrier disruption in neuromyelitis optica. *Sci Transl Med* **9**, (2017).

99. A. Iannaccone, F. Giorgianni, D. D. New, T. J. Hollingsworth, A. Umfress, A. H. Alhatem, I. Neeli, N. I. Lenchik, B. J. Jennings, J. I. Calzada, S. Satterfield, D. Mathews, R. I. Diaz, T. Harris, K. C. Johnson, S. Charles, S. B. Kritchevsky, I. C. Gerling, S. Beranova-Giorgianni, M. Z. Radic, A. B. C. s. Health, Circulating Autoantibodies in Age-Related Macular Degeneration Recognize Human Macular Tissue Antigens Implicated in Autophagy, Immunomodulation, and Protection from Oxidative Stress and Apoptosis. *PloS one* **10**, e0145323 (2015).

100. I. Korneeva, A. M. Bongiovanni, M. Girotra, T. A. Caputo, S. S. Witkin, Serum antibodies to the 27-kd heat shock protein in women with gynecologic cancers. *Am J Obstet Gynecol* **183**, 18–21 (2000).

101. H. Fillit, S. Shibata, T. Sasaki, H. Spiera, L. D. Kerr, M. Blake, Autoantibodies to the protein core of vascular basement membrane heparan sulfate proteoglycan in systemic lupus erythematosus. *Autoimmunity* **14**, 243–249 (1993).

102. H. D. Bremer, N. Landegren, R. Sjoberg, A. Hallgren, S. Renneker, E. Lattwein, D. Leonard, M. L. Eloranta, L. Ronnblom, G. Nordmark, P. Nilsson, G. Andersson, I. Lilliehook, K. Lindblad-Toh, O. Kampe, H. Hansson-Hamlin, ILF2 and ILF3 are autoantigens in canine systemic autoimmune disease. *Sci Rep* **8**, 4852 (2018).

103. S. Presslauer, G. Hinterhuber, K. Cauza, R. Horvat, K. Rappersberger, K. Wolff, D. Foedinger, RasGAPlike protein IQGAP1 is expressed by human keratinocytes and recognized by autoantibodies in association with bullous skin disease. *The Journal of investigative dermatology* **120**, 365–371 (2003).

104. T. O. Ola, P. A. Biro, M. I. Hawa, J. Ludvigsson, M. Locatelli, M. A. Puglisi, G. F. Bottazzo, A. Fierabracci, Importin beta: a novel autoantigen in human autoimmunity identified by screening random peptide libraries on phage. *J Autoimmun* **26**, 197–207 (2006).

105. Y. Lu, P. Ye, S. L. Chen, E. M. Tan, E. K. Chan, Identification of kinectin as a novel Behçet’s disease autoantigen. *Arthritis research & therapy* **7**, R1133–1139 (2005).

106. J. Inagaki, A. Kondo, L. R. Lopez, Y. Shoenfeld, E. Matsuura, Pregnancy loss and endometriosis: pathogenic role of anti-laminin-1 autoantibodies. *Annals of the New York Academy of Sciences* **1051**, 174–184 (2005).

107. C. J. Peutz-Kootstra, K. Hansen, E. De Heer, C. K. Abrass, J. A. Bruijn, Differential expression of laminin chains and anti-laminin autoantibodies in experimental lupus nephritis. *The Journal of pathology* **192**, 404–412 (2000).

108. K. Ueda, T. Nakanishi, A. Shimizu, T. Takubo, N. Matsuura, Identification of L-plastin autoantibody in plasma of patients with non-Hodgkin’s lymphoma using a proteomics-based analysis. *Ann Clin Biochem* **45**, 65–69 (2008).

109. D. Lutomski, R. Joubert-Caron, C. Lefebure, J. Salama, C. Belin, D. Bladier, M. Caron, Anti-galectin-1 autoantibodies in serum of patients with neurological diseases. *Clin Chim Acta* **262**, 131–138 (1997).

110. K. N. Konstantinov, Z. Galcheva-Gargova, M. Hoier-Madsen, A. Wiik, S. Ullman, P. Halberg, G. L. Vejlsgaard, Autoantibodies to lamins A and C in sera of patients showing peripheral fluorescent antinuclear antibody pattern on HEP-2 cells. *The Journal of investigative dermatology* **95**, 304–308 (1990).

111. A. von Mikecz, K. Konstantinov, D. S. Buchwald, L. Gerace, E. M. Tan, High frequency of autoantibodies to insoluble cellular antigens in patients with chronic fatigue syndrome. *Arthritis and rheumatism* **40**, 295–305 (1997).

112. J. Brito, G. Biamonti, R. Caporali, C. Montecucco, Autoantibodies to human nuclear lamin B2 protein. Epitope specificity in different autoimmune diseases. *Journal of immunology (Baltimore, Md. : 1950)* **153**, 2268–2277 (1994).

113. M. Tanaka, M. Kishimura, S. Ozaki, F. Osakada, H. Hashimoto, M. Okubo, M. Murakami, K. Nakao, Cloning of novel soluble gp130 and detection of its neutralizing autoantibodies in rheumatoid arthritis. *The Journal of clinical investigation* **106**, 137–144 (2000).

114. A. Gadoth, T. J. Kryzer, J. Fryer, A. McKeon, V. A. Lennon, S. J. Pittock, Microtubule-associated protein 1B: Novel paraneoplastic biomarker. *Ann Neurol* **81**, 266–277 (2017).

115. K. Suzuki, T. Nagao, M. Itabashi, Y. Hamano, R. Sugamata, Y. Yamazaki, W. Yumura, S. Tsukita, P. C. Wang, T. Nakayama, K. Suzuki, A novel autoantibody against moesin in the serum of patients with MPO-ANCA-associated vasculitis. *Nephrol Dial Transplant* **29**, 1168–1177 (2014).

116. D. Marinou, G. Katsifis, G. Barouta, C. Liaskos, L. I. Sakkas, A. Tsakris, J. G. Routsias, Major vault protein/lung resistance related protein: a novel biomarker for rheumatoid arthritis. *Clinical and experimental rheumatology*, (2020).

117. C. A. von Muhlen, E. K. Chan, C. L. Peebles, H. Imai, K. Kiyosawa, E. M. Tan, Non-muscle myosin as target antigen for human autoantibodies in patients with hepatitis C virus-associated chronic liver diseases. *Clinical and experimental immunology* **100**, 67–74 (1995).

118. B. A. Zasońska, H. Hlídková, E. Petrovský, S. Myronovskij, T. Nehrych, N. Negrych, M. Shorobura, V. Antonyuk, R. Stoika, Y. Kit, D. Horák, Monodisperse magnetic poly(glycidyl methacrylate) microspheres for isolation of autoantibodies with affinity for the 46 kDa form of unconventional Myo1C present in autoimmune patients. *Mikrochimica acta* **185**, 262 (2018).

119. R. Mossabeb, S. Seiberler, I. Mittermann, R. Reininger, S. Spitzauer, S. Natter, P. Verdino, W. Keller, D. Kraft, R. Valenta, Characterization of a novel isoform of alpha-nascent polypeptide-associated complex as IgE-defined autoantigen. *The Journal of investigative dermatology* **119**, 820–829 (2002).

120. I. N. Batova, R. T. Richardson, E. E. Widgren, M. G. O’Rand, Analysis of the autoimmune epitopes on human testicular NASP using recombinant and synthetic peptides. *Clinical and experimental immunology* **121**, 201–209 (2000).

121. Z. Qin, B. Lavingia, Y. Zou, P. Stastny, Antibodies against nucleolin in recipients of organ transplants. *Transplantation* **92**, 829–835 (2011).

122. K. Cortés-Sarabia, C. Rodríguez-Nava, Y. Medina-Flores, O. Mata-Ruíz, J. E. López-Meza, M. D. Gómez-Cervantes, I. Parra-Rojas, B. Illades-Aguiar, E. Flores-Alfaro, A. Vences-Velázquez, Production and characterization of a monoclonal antibody against the sialidase of Gardnerella vaginalis using a synthetic peptide in a MAP8 format. *Appl Microbiol Biotechnol* **104**, 6173–6183 (2020).

123. F. Le Naour, F. Brichory, D. E. Misek, C. Brechot, S. M. Hanash, L. Beretta, A distinct repertoire of autoantibodies in hepatocellular carcinoma identified by proteomic analysis. *Molecular & cellular proteomics : MCP* **1**, 197–203 (2002).

124. J. R. Underwood, X. F. Csar, B. A. Veitch, M. T. Hearn, Characterization of the specificity of a naturally-occurring monoclonal anti-thymocyte autoantibody derived from an unimmunized, neonatal Balb/c mouse. *Thymus* **21**, 199–219 (1993).

125. B. Brankin, T. C. Skaar, M. Brotzman, B. Trock, R. Clarke, Autoantibodies to the nuclear phosphoprotein nucleophosmin in breast cancer patients. *Cancer Epidemiol Biomarkers Prev* **7**, 1109–1115 (1998).

126. L. E. Andrade, E. K. Chan, C. L. Peebles, E. M. Tan, Two major autoantigen-antibody systems of the mitotic spindle apparatus. *Arthritis and rheumatism* **39**, 1643–1653 (1996).

127. R. L. Ochs, T. W. Stein, Jr., E. K. Chan, M. Ruutu, E. M. Tan, cDNA cloning and characterization of a novel nucleolar protein. *Molecular biology of the cell* **7**, 1015–1024 (1996).

128. S. Nagayama, T. Yokoi, H. Tanaka, Y. Kawaguchi, T. Shirasaka, T. Kamataki, Occurrence of autoantibody to protein disulfide isomerase in patients with hepatic disorder. *J Toxicol Sci* **19**, 163169 (1994).

129. A. Becker, N. Ludwig, A. Keller, B. Tackenberg, C. Eienbroker, W. H. Oertel, K. Fassbender, E. Meese, K. Ruprecht, Myasthenia gravis: analysis of serum autoantibody reactivities to 1827 potential human autoantigens by protein macroarrays. *PloS one* **8**, e58095 (2013).

130. A. K. Houng, L. Maggini, C. Y. Clement, G. L. Reed, Identification and structure of activated-platelet protein-1, a protein with RNA-binding domain motifs that is expressed by activated platelets. *European journal of biochemistry* **243**, 209–218 (1997).

131. K. Kaneda, Y. Takasaki, K. Takeuchi, H. Yamada, M. Nawata, M. Matsushita, R. Matsudaira, K. Ikeda, K. Yamanaka, H. Hashimoto, Autoimmune response to proteins of proliferating cell nuclear antigen multiprotein complexes in patients with connective tissue diseases. *The Journal of rheumatology* **31**, 2142–2150 (2004).

132. C. Caorsi, E. Niccolai, M. Capello, R. Vallone, M. S. Chattaragada, B. Alushi, A. Castiglione, G. Ciccone, A. Mautino, P. Cassoni, L. De Monte, S. M. Álvarez-Fernández, A. Amedei, M. Alessio, F. Novelli, Protein disulfide isomerase A3-specific Th1 effector cells infiltrate colon cancer tissue of patients with circulating anti-protein disulfide isomerase A3 autoantibodies. *Transl Res* **171**, 17–28.e11–12 (2016).

133. D. C. Chang, P. Piaggi, R. L. Hanson, W. C. Knowler, C. Bogardus, J. Krakoff, Autoantibodies against PFDN2 are associated with an increased risk of type 2 diabetes: A case-control study. *Diabetes Metab Res Rev* **33**, (2017).

134. P. J. Orchard, D. R. Nascene, A. Gupta, M. E. Taisto, L. Higgins, T. W. Markowski, T. C. Lund, Cerebral adrenoleukodystrophy is associated with loss of tolerance to profilin. *Eur J Immunol* **49**, 947–953 (2019).

135. G. Frampton, S. Moriya, J. D. Pearson, D. A. Isenberg, F. J. Ward, T. A. Smith, A. Panayiotou, N. A. Staines, J. J. Murphy, Identification of candidate endothelial cell autoantigens in systemic lupus erythematosus using a molecular cloning strategy: a role for ribosomal P protein P0 as an endothelial cell autoantigen. *Rheumatology (Oxford, England)* **39**, 1114–1120 (2000).

136. M. Wieczorek, A. Czernik, Paraneoplastic pemphigus: a short review. *Clin Cosmet Investig Dermatol* **9**, 291–295 (2016).

137. A. Kratz, M. W. Harding, J. Craft, C. G. Mackworth-Young, R. E. Handschumacher, Autoantibodies against cyclophilin in systemic lupus erythematosus and Lyme disease. *Clinical and experimental immunology* **90**, 422–427 (1992).

138. L. H. Lin, Y. W. Xu, L. S. Huang, C. Q. Hong, T. T. Zhai, L. D. Liao, W. J. Lin, L. Y. Xu, K. Zhang, E. M. Li, Y. H. Peng, Serum proteomic-based analysis identifying autoantibodies against PRDX2 and PRDX3 as potential diagnostic biomarkers in nasopharyngeal carcinoma. *Clin Proteomics* **14**, 6 (2017).

139. S. Kobayashi, T. Hiwasa, T. Arasawa, A. Kagaya, S. Ishii, H. Shimada, M. Ito, M. Suzuki, M. Kano, B. Rahmutulla, K. Kitamura, Y. Sawabe, H. Shin, M. Takiguchi, F. Nomura, H. Matsubara, K. Matsushita, Identification of specific and common diagnostic antibody markers for gastrointestinal cancers by SEREX screening using testis cDNA phage library. *Oncotarget* **9**, 18559–18569 (2018).

140. C. Schild-Poulter, A. Su, A. Shih, O. P. Kelly, M. J. Fritzler, R. Goldstein, R. J. Hache, Association of autoantibodies with Ku and DNA repair proteins in connective tissue diseases. *Rheumatology (Oxford, England)* **47**, 165–171 (2008).

141. E. Feist, U. Kuckelkorn, T. Dörner, H. Dönitz, S. Scheffler, F. Hiepe, P. M. Kloetzel, G. R. Burmester, Autoantibodies in primary Sjögren’s syndrome are directed against proteasomal subunits of the alpha and beta type. *Arthritis and rheumatism* **42**, 697–702 (1999).

142. E. Feist, T. Dorner, U. Kuckelkorn, G. Schmidtke, B. Micheel, F. Hiepe, G. R. Burmester, P. M. Kloetzel, Proteasome alpha-type subunit C9 is a primary target of autoantibodies in sera of patients with myositis and systemic lupus erythematosus. *The Journal of experimental medicine* **184**, 1313–1318 (1996).

143. C. Bohring, W. Krause, Characterization of spermatozoa surface antigens by antisperm antibodies and its influence on acrosomal exocytosis. *Am J Reprod Immunol* **50**, 411–419 (2003).

144. K. Sugimoto, T. Hiwasa, K. Shibuya, S. Hirano, M. Beppu, S. Isose, K. Arai, M. Takiguchi, S. Kuwabara, M. Mori, Novel autoantibodies against the proteasome subunit PSMA7 in amyotrophic lateral sclerosis. *J Neuroimmunol* **325**, 54–60 (2018).

145. S. Scheffler, U. Kuckelkorn, K. Egerer, T. Dörner, K. Reiter, A. Soza, G. R. Burmester, E. Feist, Autoimmune reactivity against the 20S-proteasome includes immunosubunits LMP2 (beta1i), MECL1 (beta2i) and LMP7 (beta5i). *Rheumatology (Oxford, England)* **47**, 622–626 (2008).

146. Z. Mojtahedi, A. Safaei, Z. Yousefi, A. Ghaderi, Immunoproteomics of HER2-positive and HER2negative breast cancer patients with positive lymph nodes. *OMICS* **15**, 409–418 (2011).

147. C. Montecucco, R. Caporali, F. Cobianchi, G. Biamonti, Identification of autoantibodies to the I protein of the heterogeneous nuclear ribonucleoprotein complex in patients with systemic sclerosis. *Arthritis and rheumatism* **39**, 1669–1676 (1996).

148. D. F. Fiorentino, M. Presby, A. N. Baer, M. Petri, K. E. Rieger, M. Soloski, A. Rosen, A. L. Mammen, L. Christopher-Stine, L. Casciola-Rosen, PUF60: a prominent new target of the autoimmune response in dermatomyositis and Sjögren’s syndrome. *Annals of the rheumatic diseases* **75**, 1145–1151 (2016).

149. I. Schepens, F. Jaunin, N. Begre, U. Läderach, K. Marcus, T. Hashimoto, B. Favre, L. Borradori, The protease inhibitor alpha-2-macroglobulin-like-1 is the p170 antigen recognized by paraneoplastic pemphigus autoantibodies in human. *PloS one* **5**, e12250 (2010).

150. S. Thébault, D. Gilbert, M. Hubert, L. Drouot, N. Machour, C. Lange, R. Charlionet, F. Tron, Orderly pattern of development of the autoantibody response in (New Zealand White x BXSB)F1 lupus mice: characterization of target antigens and antigen spreading by two-dimensional gel electrophoresis and mass spectrometry. *Journal of immunology (Baltimore, Md. : 1950)* **169**, 4046–4053 (2002).

151. J. H. Vaughan, J. R. Valbracht, M. D. Nguyen, H. H. Handley, R. S. Smith, K. Patrick, G. H. Rhodes, Epstein-Barr virus-induced autoimmune responses. I. Immunoglobulin M autoantibodies to proteins mimicking and not mimicking Epstein-Barr virus nuclear antigen-1. *The Journal of clinical investigation* **95**, 1306–1315 (1995).

152. K. Doe, K. Nozawa, K. Hiruma, Y. Yamada, Y. Matsuki, S. Nakano, M. Ogasawara, H. Nakano, T. Ikeda, T. Ikegami, M. Fujishiro, M. Kawasaki, K. Ikeda, H. Amano, S. Morimoto, H. Ogawa, K. Takamori, I. Sekigawa, Y. Takasaki, Antibody against chromatin assembly factor-1 is a novel autoantibody specifically recognized in systemic lupus erythematosus. *Lupus* **23**, 1031–1041 (2014).

153. M. Wagatsuma, M. Kimura, R. Suzuki, F. Takeuchi, K. Matsuta, H. Watanabe, Ezrin, radixin and moesin are possible auto-immune antigens in rheumatoid arthritis. *Molecular immunology* **33**, 1171–1176 (1996).

154. T. Sato, T. Uchiumi, R. Kominami, M. Arakawa, Autoantibodies specific for the 20-KDal ribosomal large subunit protein L12. *Biochemical and biophysical research communications* **172**, 496–502 (1990).

155. A. Guialis, M. Patrinou-Georgoula, N. Tsifetaki, V. Aidinis, C. E. Sekeris, H. M. Moutsopoulos, Anti-5S RNA/protein (RNP) antibody levels correlate with disease activity in a patient with systemic lupus erythematosus (SLE) nephritis. *Clinical and experimental immunology* **95**, 385–389 (1994).

156. E. Neu, A. H. von Mikecz, P. H. Hemmerich, H. H. Peter, M. Fricke, H. Deicher, E. Genth, U. Krawinkel, Autoantibodies against eukaryotic protein L7 in patients suffering from systemic lupus erythematosus and progressive systemic sclerosis: frequency and correlation with clinical, serological and genetic parameters. The SLE Study Group. *Clinical and experimental immunology* **100**, 198–204 (1995).

157. K. Elkon, E. Bonfa, R. Llovet, W. Danho, H. Weissbach, N. Brot, Properties of the ribosomal P2 protein autoantigen are similar to those of foreign protein antigens. *Proceedings of the National Academy of Sciences of the United States of America* **85**, 5186–5189 (1988).

158. L. Y. Luo, I. Herrera, A. Soosaipillai, E. P. Diamandis, Identification of heat shock protein 90 and other proteins as tumour antigens by serological screening of an ovarian carcinoma expression library. *British journal of cancer* **87**, 339–343 (2002).

159. M. Absi, J. P. La Vergne, A. Marzouki, F. Giraud, D. Rigal, A. M. Reboud, J. P. Reboud, J. C. Monier, Heterogeneity of ribosomal autoantibodies from human, murine and canine connective tissue diseases. *Immunology letters* **23**, 35–41 (1989).

160. K. T. Tycowski, M. D. Shu, J. A. Steitz, A small nucleolar RNA is processed from an intron of the human gene encoding ribosomal protein S3. *Genes & development* **7**, 1176–1190 (1993).

161. Z. Betteridge, H. Gunawardena, J. North, J. Slinn, N. McHugh, Identification of a novel autoantibody directed against small ubiquitin-like modifier activating enzyme in dermatomyositis. *Arthritis and rheumatism* **56**, 3132–3137 (2007).

162. A. M. Abreu-Velez, M. S. Howard, K. Hashimoto, T. Hashimoto, Autoantibodies to sweat glands detected by different methods in serum and in tissue from patients affected by a new variant of endemic pemphigus foliaceus. *Archives of dermatological research* **301**, 711–718 (2009).

163. P. Margutti, M. Sorice, F. Conti, F. Delunardo, M. Racaniello, C. Alessandri, A. Siracusano, R. Riganò, E. Profumo, G. Valesini, E. Ortona, Screening of an endothelial cDNA library identifies the C-terminal region of Nedd5 as a novel autoantigen in systemic lupus erythematosus with psychiatric manifestations. *Arthritis research & therapy* **7**, R896–903 (2005).

164. R. L. Bates, S. J. Payne, S. L. Drury, P. N. Nelson, D. A. Isenberg, J. J. Murphy, G. Frampton, The prevalence and clinical significance of autoantibodies to plasminogen activator inhibitor 1 in systemic lupus erythematosus. *Lupus* **12**, 617–622 (2003).

165. S. Yokota, H. Kubota, Y. Matsuoka, M. Naitoh, D. Hirata, S. Minota, H. Takahashi, N. Fujii, K. Nagata, Prevalence of HSP47 antigen and autoantibodies to HSP47 in the sera of patients with mixed connective tissue disease. *Biochemical and biophysical research communications* **303**, 413–418 (2003).

166. H. M. Hwang, C. K. Heo, H. J. Lee, S. S. Kwak, W. H. Lim, J. S. Yoo, D. Y. Yu, K. J. Lim, J. Y. Kim, E. W. Cho, Identification of anti-SF3B1 autoantibody as a diagnostic marker in patients with hepatocellular carcinoma. *J Transl Med* **16**, 177 (2018).

167. Y. Hosono, R. Nakashima, S. Serada, K. Murakami, Y. Imura, H. Yoshifuji, K. Ohmura, T. Naka, T. Mimori, Splicing factor proline/glutamine-rich is a novel autoantigen of dermatomyositis and associated with anti-melanoma differentiation-associated gene 5 antibody. *J Autoimmun* **77**, 116–122 (2017).

168. K. Overzet, T. J. Gensler, S. J. Kim, M. E. Geiger, W. J. van Venrooij, K. M. Pollard, P. Anderson, P. J. Utz, Small nucleolar RNP scleroderma autoantigens associate with phosphorylated serine/arginine splicing factors during apoptosis. *Arthritis and rheumatism* **43**, 1327–1336 (2000).

169. A. M. Yamamoto, Z. Amoura, C. Johannet, A. L. Jeronimo, H. Campos, S. Koutouzov, J. C. Piette, J. F. Bach, Quantitative radioligand assays using de novo-synthesized recombinant autoantigens in connective tissue diseases: new tools to approach the pathogenic significance of anti-RNP antibodies in rheumatic diseases. *Arthritis and rheumatism* **43**, 689–698 (2000).

170. J. D. Huntriss, D. S. Latchman, D. G. Williams, Lupus autoantibodies discriminate between the highly homologous Sm polypeptides B/B’ and SmN by binding an epitope restricted to B/B’. *Clinical and experimental immunology* **92**, 263–267 (1993).

171. H. Brahms, J. Raymackers, A. Union, F. de Keyser, L. Meheus, R. Luhrmann, The C-terminal RG dipeptide repeats of the spliceosomal Sm proteins D1 and D3 contain symmetrical dimethylarginines, which form a major B-cell epitope for anti-Sm autoantibodies. *The Journal of biological chemistry* **275**, 17122–17129 (2000).

172. M. T. McClain, P. A. Ramsland, K. M. Kaufman, J. A. James, Anti-sm autoantibodies in systemic lupus target highly basic surface structures of complexed spliceosomal autoantigens. *Journal of immunology (Baltimore, Md. : 1950)* **168**, 2054–2062 (2002).

173. M. Satoh, J. Y. Chan, S. J. Ross, A. Ceribelli, I. Cavazzana, F. Franceschini, Y. Li, W. H. Reeves, E. S. Sobel, E. K. Chan, Autoantibodies to survival of motor neuron complex in patients with polymyositis: immunoprecipitation of D, E, F, and G proteins without other components of small nuclear ribonucleoproteins. *Arthritis and rheumatism* **63**, 1972–1978 (2011).

174. M. Garbarz, D. Dhermy, O. Bournier, A. Bezeaud, P. Boivin, Anti-spectrin in sera containing smooth muscle autoantibodies from patients with chronic active hepatitis. *Clinical and experimental immunology* **43**, 87–93 (1981).

175. A. Zaninoni, C. Vercellati, F. G. Imperiali, A. P. Marcello, B. Fattizzo, E. Fermo, P. Bianchi, C. Grossi, A. Cattaneo, A. Cortelezzi, A. Zanella, W. Barcellini, Detection of red blood cell antibodies in mitogen-stimulated cultures from patients with hereditary spherocytosis. *Transfusion* **55**, 29302938 (2015).

176. P. Santoro, M. De Andrea, G. Migliaretti, C. Trapani, S. Landolfo, M. Gariglio, High prevalence of autoantibodies against the nuclear high mobility group (HMG) protein SSRP1 in sera from patients with systemic lupus erythematosus, but not other rheumatic diseases. *The Journal of rheumatology* **29**, 90–93 (2002).

177. A. Cortini, S. Bembich, L. Marson, E. Cocco, P. Edomi, Identification of novel non-myelin biomarkers in multiple sclerosis using an improved phage-display approach. *PloS one* **14**, e0226162 (2019).

178. Y. Meng, M. Zhang, X. Zhao, Y. Cheng, R. Jia, Y. Wang, X. Sun, Decreased serum thrombospondin-1 and elevation of its autoantibody are associated with multiple exacerbated clinical manifestations in systemic lupus erythematosus. *Clinical rheumatology* **37**, 2707–2714 (2018).

179. M. Muto, M. Mori, T. Hiwasa, M. Takiguchi, Y. Iwadate, A. Uzawa, T. Uchida, H. Masuda, K. Sugimoto, S. Kuwabara, Novel serum autoantibodies against talin1 in multiple sclerosis: Possible pathogenetic roles of the antibodies. *J Neuroimmunol* **284**, 30–36 (2015).

180. A. Schwenzer, X. Jiang, T. R. Mikuls, J. B. Payne, H. R. Sayles, A. M. Quirke, B. M. Kessler, R. Fischer, P. J. Venables, K. Lundberg, K. S. Midwood, Identification of an immunodominant peptide from citrullinated tenascin-C as a major target for autoantibodies in rheumatoid arthritis. *Annals of the rheumatic diseases* **75**, 1876–1883 (2016).

181. X. Geng, L. Biancone, H. H. Dai, J. J. Lin, N. Yoshizaki, A. Dasgupta, F. Pallone, K. M. Das, Tropomyosin isoforms in intestinal mucosa: production of autoantibodies to tropomyosin isoforms in ulcerative colitis. *Gastroenterology* **114**, 912–922 (1998).

182. R. Gajbhiye, A. Sonawani, S. Khan, A. Suryawanshi, S. Kadam, N. Warty, V. Raut, V. Khole, Identification and validation of novel serum markers for early diagnosis of endometriosis. *Hum Reprod* **27**, 408–417 (2012).

183. A. Kimura, T. Sakurai, M. Yamada, A. Koumura, Y. Hayashi, Y. Tanaka, I. Hozumi, H. Ohtaki, M. Chousa, M. Takemura, M. Seishima, T. Inuzuka, Anti-endothelial cell antibodies in patients with cerebral small vessel disease. *Curr Neurovasc Res* **9**, 296–301 (2012).

184. P. Enarson, J. B. Rattner, Y. Ou, K. Miyachi, T. Horigome, M. J. Fritzler, Autoantigens of the nuclear pore complex. *J Mol Med (Berl)* **82**, 423–433 (2004).

185. X. Zhao, Y. Cheng, Y. Gan, R. Jia, L. Zhu, X. Sun, Anti-tubulin-alpha-1C autoantibody in systemic lupus erythematosus: a novel indicator of disease activity and vasculitis manifestations. *Clinical rheumatology* **37**, 1229–1237 (2018).

186. T. Matthes, A. Wolff, P. Soubiran, F. Gros, G. Dighiero, Antitubulin antibodies. II. Natural autoantibodies and induced antibodies recognize different epitopes on the tubulin molecule. *Journal of immunology (Baltimore, Md. : 1950)* **141**, 3135–3141 (1988).

187. A. Kimura, N. Yoshikura, A. Koumura, Y. Hayashi, Y. Kobayashi, I. Kobayashi, T. Yano, T. Inuzuka, Identification of target antigens of naturally occurring autoantibodies in cerebrospinal fluid. *Journal of proteomics* **128**, 450–457 (2015).

188. L. Prasannan, D. E. Misek, R. Hinderer, J. Michon, J. D. Geiger, S. M. Hanash, Identification of beta-tubulin isoforms as tumor antigens in neuroblastoma. *Clin Cancer Res* **6**, 3949–3956 (2000).

189. Y. Muro, Y. Ogawa, Y. Kato, M. Hagiwara, Autoantibody to thioredoxin reductase in an ovarian cancer patient. *Biochemical and biophysical research communications* **242**, 267–271 (1998).

190. Z. E. Betteridge, H. Gunawardena, H. Chinoy, J. North, W. E. Ollier, R. G. Cooper, N. J. McHugh, U. K. A. O. M. I. Collaboration, Clinical and human leucocyte antigen class II haplotype associations of autoantibodies to small ubiquitin-like modifier enzyme, a dermatomyositis-specific autoantigen target, in UK Caucasian adult-onset myositis. *Annals of the rheumatic diseases* **68**, 1621–1625 (2009).

191. X. Li, J. Sun, R. Mu, Y. Gan, G. Wang, J. He, L. Yi, Q. Wang, X. Sun, Z. Li, The clinical significance of ubiquitin carboxyl hydrolase L1 and its autoantibody in neuropsychiatric systemic lupus erythematosus. *Clinical and experimental rheumatology* **37**, 474–480 (2019).

192. Y. Zhou, J. Cui, H. Du, Autoantibody-targeted TAAs in pancreatic cancer: A comprehensive analysis. *Pancreatology* **19**, 760–768 (2019).

193. K. Miyachi, H. Hosaka, N. Nakamura, H. Miyakawa, T. Mimori, M. Shibata, S. Matsushima, H. Chinoh, T. Horigome, R. W. Hankins, M. Zhang, M. J. Fritzler, Anti-p97/VCP antibodies: an autoantibody marker for a subset of primary biliary cirrhosis patients with milder disease? *Scand J Immunol* **63**, 376–382 (2006).

194. F. J. Li, R. Surolia, H. Li, Z. Wang, T. Kulkarni, G. Liu, J. A. de Andrade, D. J. Kass, V. J. Thannickal, S. R. Duncan, V. B. Antony, Autoimmunity to Vimentin Is Associated with Outcomes of Patients with Idiopathic Pulmonary Fibrosis. *Journal of immunology (Baltimore, Md. : 1950)* **199**, 1596–1605 (2017).

195. E. L. Paley, N. Alexandrova, L. Smelansky, Tryptophanyl-tRNA synthetase as a human autoantigen. *Immunology letters* **48**, 201–207 (1995).

196. T. Mimori, Y. Ohosone, N. Hama, A. Suwa, M. Akizuki, M. Homma, A. J. Griffith, J. A. Hardin, Isolation and characterization of cDNA encoding the 80-kDa subunit protein of the human autoantigen Ku (p70/p80) recognized by autoantibodies from patients with scleroderma-polymyositis overlap syndrome. *Proceedings of the National Academy of Sciences of the United States of America* **87**, 1777–1781 (1990).

197. S. Hoa, M. Hudson, Y. Troyanov, S. Proudman, J. Walker, W. Stevens, M. Nikpour, S. Assassi, M. D. Mayes, M. Wang, M. Baron, M. J. Fritzler, G. Canadian Scleroderma Research, G. Australian Scleroderma Interest, S. Genetics versus Environment in Scleroderma Outcome, Single-specificity anti-Ku antibodies in an international cohort of 2140 systemic sclerosis subjects: clinical associations. *Medicine (Baltimore)* **95**, e4713 (2016).

198. D. Braunschweig, P. Krakowiak, P. Duncanson, R. Boyce, R. L. Hansen, P. Ashwood, I. Hertz-Picciotto, I. N. Pessah, J. Van de Water, Autism-specific maternal autoantibodies recognize critical proteins in developing brain. *Transl Psychiatry* **3**, e277 (2013).

199. A. Kistner, M. B. Bigler, K. Glatz, S. B. Egli, F. S. Baldin, F. A. Marquardsen, M. Mehling, K. M. Rentsch, D. Staub, M. Aschwanden, M. Recher, T. Daikeler, C. T. Berger, Characteristics of autoantibodies targeting 14-3-3 proteins and their association with clinical features in newly diagnosed giant cell arteritis. *Rheumatology (Oxford, England)* **56**, 829–834 (2017).

200. M. H. van Beers-Tas, A. Marotta, M. Boers, W. P. Maksymowych, D. van Schaardenburg, A prospective cohort study of 14-3-3eta in ACPA and/or RF-positive patients with arthralgia. *Arthritis research & therapy* **18**, 76 (2016).

201. J. Qiu, G. Choi, L. Li, H. Wang, S. J. Pitteri, S. R. Pereira-Faca, A. L. Krasnoselsky, T. W. Randolph, G. S. Omenn, C. Edelstein, M. J. Barnett, M. D. Thornquist, G. E. Goodman, D. E. Brenner, Z. Feng, S. M. Hanash, Occurrence of autoantibodies to annexin I, 14-3-3 theta and LAMR1 in prediagnostic lung cancer sera. *J Clin Oncol* **26**, 5060–5066 (2008).

202. R. Chakravarti, K. Gupta, M. Swain, B. Willard, J. Scholtz, L. G. Svensson, E. E. Roselli, G. Pettersson, D. R. Johnston, E. G. Soltesz, M. Yamashita, D. Stuehr, T. M. Daly, G. S. Hoffman, 14-3-3 in Thoracic Aortic Aneurysms: Identification of a Novel Autoantigen in Large Vessel Vasculitis. *Arthritis Rheumatol* **67**, 1913–1921 (2015).

Notes: # Pep., number of peptides identified by mass spectrometry; COVID (Up/Down), protein or gene expression up- and/or down-regulated in SARS-Cov-2 infected cells or patients; DS affinity, concentration of NaCl (1.0 M, very high affinity, or 0.5 M, medium to high affinity) at which a DS-binding protein elutes from DS-affinity resin.

**Table 2.** Top enriched pathways and processes related to COVID-altered proteins

COVID	Ontology	Description	Count	%	Log_10_(P)
**Altered**	R-HSA-8953854	Metabolism of RNA	78	22.16	−51.2
	R-HSA-422475	Axon guidance	63	17.90	−40.6
	GO:0006412	Translation	66	18.75	−35.9
	GO:0000377	RNA splicing	44	12.50	−28.0
	GO:0045055	Regulated exocytosis	58	16.48	−26.7
	GO:0006457	Protein folding	33	9.38	−24.3
	R-HSA-1474244	Extracellular matrix organization	33	9.38	−20.6
	GO:0043687	Post-translational protein modification	35	9.94	−20.0
	GO:0071826	Ribonucleoprotein complex subunit organization	32	9.09	−19.7
	CORUM:5615	Emerin complex 52	13	3.69	−18.8
	GO:0010638	Positive regulation of organelle organization	40	11.36	−16.1
	GO:0042060	Wound healing	38	10.80	−15.6
	GO:0006913	Nucleocytoplasmic transport	30	8.52	−15.6
	R-HSA-114608	Platelet degranulation	19	6.27	−15.6
	R-HSA-5653656	Vesicle-mediated transport	40	11.36	−15.4
	GO:0097435	Supramolecular fiber organization	41	11.65	−15.1
	CORUM:1335	SNW1 complex	10	3.30	−15.1
	GO:0002181	Cytoplasmic translation	18	5.11	−15.1
	R-HSA-445355	Smooth Muscle Contraction	13	3.69	−14.9
	GO:0031647	Regulation of protein stability	27	7.67	−14.9
Up	R-HSA-72163	mRNA Splicing - Major Pathway	23	8.85	−18.8
	R-HSA-449147	Signaling by Interleukins	26	10.00	−12.6
	GO:0000904	Cell morphogenesis involved in differentiation	31	11.92	−11.3
Down	R-HSA-2262752	Cellular responses to stress	55	18.15	−34.5
	R-HSA-109581	Apoptosis	23	7.59	−17.5
	GO:0035966	Response to topologically incorrect protein	22	7.26	−15.0

Count: the number of DS-affinity proteins with membership in the given ontology term. %: percentage of DS-affinity proteins in the given ontology term.
